# NR1D1 deficiency in the tumor microenvironment promotes lung tumor development by activating the NLRP3 inflammasome

**DOI:** 10.1038/s41420-023-01554-3

**Published:** 2023-07-31

**Authors:** Sun Mi Kim, Yoon Jeon, Ji Yun Jang, Ho Lee

**Affiliations:** 1 Graduate School of Cancer Science and Policy, National Cancer Center, Gyeonggi, 10408 Republic of Korea; 2 Research Institute, National Cancer Center, Gyeonggi, 10408 Republic of Korea; 3 College of Pharmacy, Dongguk University, Seoul, 04620 Republic of Korea

**Keywords:** Lung cancer, Tumour-suppressor proteins

## Abstract

Nuclear receptor Rev-erbα (NR1D1) is a major negative regulator of the circadian clock. Numerous studies have investigated the role of circadian clock-related factors in the tumorigenesis of multiple cancer types, but little is known about the role of NR1D1 in cancer development. In this study, we identified the role of NR1D1 in lung tumorigenesis using genetically engineered mouse models of *Nr1d1*. Although NR1D1 overexpression or knockdown had little effect on the proliferation of NSCLC cells in vitro, NR1D1 deficiency in the tumor microenvironment increased lung cancer development compared with the control in the orthotopic model. NR1D1-deficient mice showed increased NACHT, LRR, and PYD domain-containing protein 3 (NLRP3) inflammasome activation, and conditioned medium (CM) from NR1D1-deficient macrophages increased the proliferation and epithelial–mesenchymal transition (EMT) of lung cancer cells. Treatment with MCC950, a specific inhibitor of NLRP3 inflammasome, blocked tumorigenesis in NR1D1-deficient mice in an orthotopic lung cancer model. In addition, MCC950 treatment blocked the increased proliferation and EMT of cancer cells induced by CM from NR1D1-deficient macrophages in vitro. Our results showed that NR1D1 in the tumor microenvironment functions as a tumor suppressor by negatively regulating the NLRP3 inflammasome, suggesting that the NLRP3 inflammasome blockade via NR1D1 activation could be a therapeutic strategy to overcome lung cancer.

## Introduction

Lung cancer is one of the most common cancers and has a high mortality rate, accounting for 18% of cancer-related deaths in 2020 [1]. Although the survival rate of lung cancer patients has increased through the continuous development of several therapeutic methods, such as chemotherapy, targeted therapy, and immunotherapy, the 5-year survival rate of lung cancer patients is only approximately 15%, suggesting that there is still a limitation to overcome lung cancer [2, 3]. Therefore, to overcome lung cancer and improve the survival rate of patients, it is urgent to find new therapeutic targets.

The tumor microenvironment (TME) comprises many stromal cells, such as various immune cells, fibroblasts, and vascular endothelial cells. These stromal cells are well known to play a critical role in promoting cancer development and metastasis by interacting with cancer cells [4]. Studies on the effect of TME on lung cancer occurrence, metastasis, and efficacy of anti-cancer therapy for lung cancer are steadily progressing, and TME has been suggested as an attractive therapeutic target for lung cancer treatment [5].

The circadian clock is an internal clock that recognizes environmental signals, such as light and food in most living organisms, to govern various physiological and biochemical processes, such as sleeping, food intake, body temperature regulation, and hormone secretion [6, 7]. Disruption of the circadian clock causes systemic dysfunction and promotes the pathogenesis of diseases, such as impaired immune function, metabolic disorders including obesity or diabetes, and cancer [6]. Numerous studies have highlighted the role of the circadian clock in tumorigenesis. Mice with genetic disruptions of *Per2*, *Bmal1*, *Cry1*, and *Cry2*, which are regulatory factors of the circadian clock, spontaneously developed cancer in multiple organs, including the liver and ovaries [8, 9]. Additionally, the loss of the *Per2* gene promoted tumorigenesis in *Apc*^*min/+*^ mouse colon cancer and *Kras*^*G12D*^ mouse lung cancer models [10, 11]. *Bmal1* deletion also increased tumor incidence in the *Kras*^*G12D*^ mouse lung cancer model [11]. On the other hand, several studies showed that core clock genes function as an oncogene. CLOCK and BMAL1 were necessary for cell proliferation and stemness maintenance in acute myeloid leukemia [12]. In addition, it was reported that a loss of the *Cry1* and *Cry2* genes suppresses tumor development in *p53* mutant mice [13].

Nuclear receptor Rev-erbα (NR1D1) is a key regulatory factor leading the negative feedback loop of the circadian clock [14]. It was reported that NR1D1 negatively regulates rheumatoid arthritis and fulminant hepatitis through the inhibition of inflammation and suppresses asthma through blockade of Th2 cell differentiation [15–17]. Based on previous reports that the major factors of the circadian clock are closely related to tumorigenesis, NR1D1 is also expected to affect tumor development. In fact, several studies reported the role of NR1D1 in tumorigenesis. Synthetic NR1D1/2 agonists, such as SR9009 and SR9011, showed cell cytotoxicity in cancer cells derived from glioblastoma, leukemia, colon cancer, and melanoma [18]. Also, SR9011 was found to inhibit the growth of breast cancer cells regardless of breast cancer subtypes [19]. Similar to the agonists, NR1D1 overexpression inhibited proliferation and induced apoptosis in ovarian cancer cells [20]. Although several reports suggest that NR1D1 functions as a tumor suppressor, its role of NR1D1 in lung cancer development has not been clearly elucidated.

NACHT, LRR and PYD domains-containing protein 3 (NLRP3) inflammasome plays a critical role in the innate immune system [21]. The active NLRP3 inflammasome, composed of apoptosis-associated speck-like protein containing a caspase-recruitment domain (ASC) and caspase-1, induces the maturation of interleukin 1β (IL1β) and 18 (IL18), resulting in a strong pro-inflammatory response and subsequent immune responses [21, 22]. Recently, it has been reported that the NLRP3 inflammasome is closely related to the development of several types of cancer. Several groups have reported that the NLRP3 inflammasome plays a positive role in tumorigenesis and metastasis in lung, breast, or gastric cancers [23–26]. Conversely, there is also a report expressing that the NLRP3 inflammasome acts as a tumor suppressor in azoxymethane/dextran sulfate sodium-induced colitis-associated colorectal cancer [27]. Although the role of NLRP3 inflammasome in cancer development is controversial, it is perceived as an attractive therapeutic target for cancer treatment.

In this study, we analyzed the role of NR1D1 in lung tumorigenesis using genetically engineered mouse models. Deletion or overexpression of NR1D1 in the tumor microenvironment increased or reduced lung tumor development in an NLRP3 inflammasome-dependent manner, suggesting that NR1D1 acts as a tumor suppressor in the lung tumor microenvironment.

## Results

### NR1D1 deficiency in mice promotes lung tumor development

**Fig. 1:**
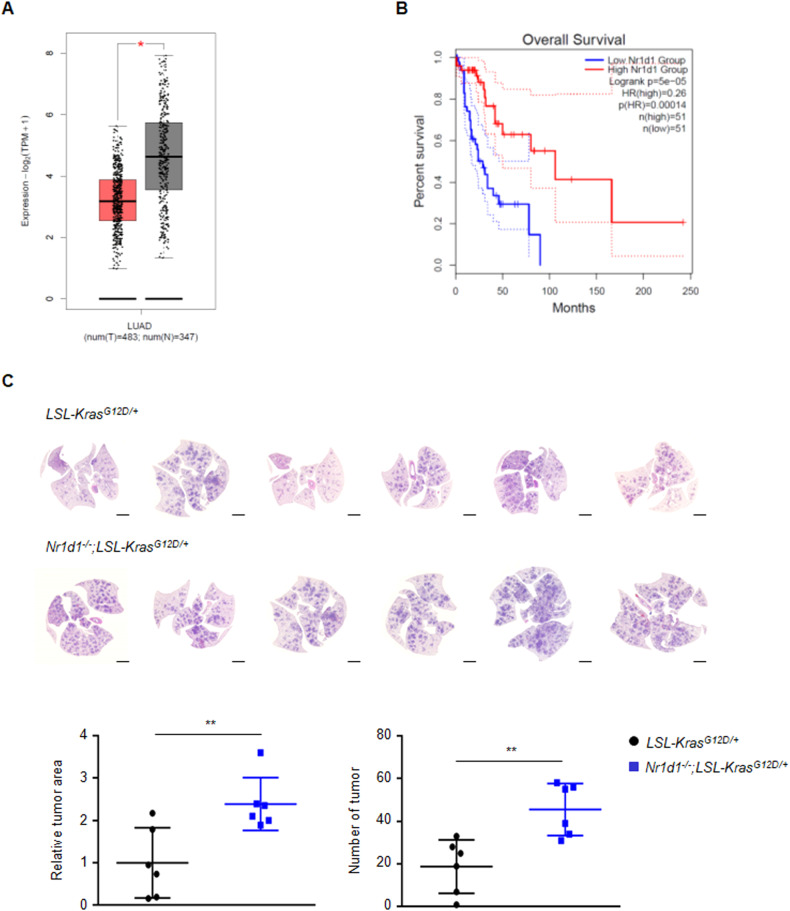
NR1D1 deficiency promotes tumorigenesis in mouse lung cancer models. **A** GEPIA2 data analysis for *NR1D1* expression in normal and lung adenocarcinomas (LUAD). The red and gray boxes indicate tumor (*n* = 483) and normal (*n* = 347) samples, respectively. *p < 0.01. **B** The overall survival rate of human LUAD patients in relation to high or low expression levels of *NR1D1*. Solid line=survival rate; dotted line=95% confidence interval; HR (Hazards Ratio)=0.28. **C** The upper panel shows images from H&E staining for the lungs of *Kras*^*G12D/+*^ and *Nr1d1*^*-/-*^*;Kras*^*G12D/+*^ mice 8 weeks after AdenoCre virus inhalation. Scale bars, 2 mm. The lower panel indicates the relative tumor area (normalized to total lung area) and the number of tumor between *Kras*^*G12D/+*^ (*n* = 6) and *Nr1d1*^*-/-*^*;Kras*^*G12D/+*^ (*n* = 6) mice. The results are expressed as the mean ± SD. **p < 0.01.

Before investigating the role of NR1D1 in cancer development, we analyzed the expression of *NR1D1* mRNA in various cancer types from TCGA and GTEx databases using GEPIA2. As a result of analysis in 33 cancer types, *NR1D1* mRNA levels were significantly lower in cancer tissues than in normal tissues in 10 cancer types, including LUAD. The opposite expression pattern was observed in the three cancer types (Figs. 1A and S1). In cancer types with a significant difference in *NR1D1* expression between cancer and normal tissues, the correlation between the expression level of *NR1D1* and survival rate was analyzed. In these analyses, LUAD showed a significant correlation between the expression level of *NR1D1* and the survival rate (Fig. 1B). Consistent with this result, a previous report showed that *NR1D1* levels were lower in LUAD tissues than in the corresponding normal tissues in LUAD patients [28]. Based on these analyses, we investigated whether NR1D1 plays a tumor suppressive role in lung tumorigenesis.

First, we generated new *Nr1d1* KO mice using the CRISPR/Cas9 system. As previously reported, *Nr1d1*^*−/−*^ mice derived from the *Nr1d1*^*tm1Ics*^ allele unexpectedly expressed truncated NR1D1 protein in the cytoplasm [29]. The newly developed *Nr1d1*^*−/−*^ mice did not express any NR1D1 protein (Fig. S2). We then crossed *Nr1d1*^*−/−*^ mice with *LSL-Kras*^*G12D/+*^ mice to identify the role of NR1D1 in lung tumor development [30]. As shown in Fig. 1C, *Nr1d1*^*−/−*^ mice showed an approximately 2.5-fold increase in Kras-driven lung tumor development compared to WT mice.

To prove the tumor suppressive role of NR1D1 in vitro, we investigated whether NR1D1 overexpression (OE) induces cell cycle arrest or apoptosis in non-small cell lung cancer (NSCLC) cell lines (A549, H358, and H1299). Although NR1D1 was successfully overexpressed in all three cell lines, we unexpectedly did not observe cell cycle arrest (Fig. S3A, B). In addition, NR1D1 OE-induced apoptosis was barely detectable in any of the three NSCLC cell lines (Fig. S3C). Additionally, we did not observe an alteration in the cell cycle distribution in NSCLC cells upon the knockdown (KD) of *NR1D1* (Fig. S3D, E). These results suggest the possibility that NR1D1 may play a role in the lung TME rather than the cancer cells themselves.

### NR1D1 plays a tumor suppressive role in the lung tumor microenvironment

**Fig. 2:**
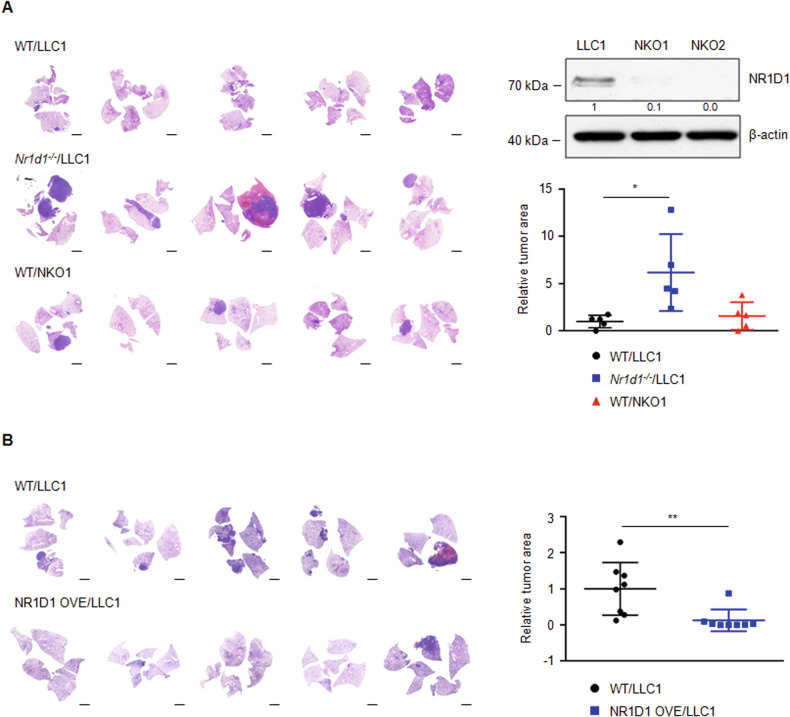
NR1D1 plays a tumor suppressive role in the tumor microenvironment. **A** The left panel shows images from H&E staining for the lungs of LLC1-injected WT or *Nr1d1*^*−/−*^ mice and NKO1-injected WT mice. Scale bars, 2 mm. The upper right panel shows western blot analysis for NR1D1 expression in NR1D1-deficient LLC1 clones (NKO1 and NKO2). The lower right panel indicates the relative tumor area (normalized to total lung) between groups (*n* = 5 in each group). The results are expressed as the mean ± SD. *p < 0.05. **B** The left panel shows representative images from H&E staining for lung of LLC1-injected WT or NR1D1 OVE mice. Scale bars, 2 mm. The right panel shows the relative tumor area (normalized to total lung area) between WT (*n* = 8) or NR1D1 OVE (*n* = 8) mice. The results are expressed as the mean ± SD. **p < 0.01.

To examine whether NR1D1 has a tumor suppressive role in the lung TME through in vivo experiments, mouse Lewis lung carcinoma cells (LLC1) were orthotopically injected into the lungs of syngeneic WT or *Nr1d1*-null mice [31]. The total lung tumor area in LLC1-injected *Nr1d1*^*−/−*^ mice was approximately six-fold greater than that in LLC1-injected WT mice (Fig. 2A).

Subsequently, to investigate the effect of NR1D1 deficiency in LLC1 cells, we generated NR1D1*-*deficient LLC1 cell clone (NKO1 and NKO2) using the CRISPR/Cas9 system and confirmed the NR1D1 deficiency in the NKO1 and NKO2 cells using western blot analysis (Fig. 2A). Then, NKO1 and NKO2 cells were orthotopically injected into the lungs of syngeneic WT mice. Interestingly, the total lung tumor area in NKO1-injected mice was slightly increased compared with that in the LLC1-injected mice, but the difference was not significant (Fig. 2A). Additionally, NKO2-injected mice showed lung tumor development similar to that of the LLC1-injected mice, which was consistent with the results of NKO1-injected mice (Fig. S4). To confirm the tumor suppressive role of NR1D1, tamoxifen-inducible NR1D1 overexpressing mice (NR1D1 OVE) were generated by crossing *Nr1d1* transgenic mice with *Cre*^*ERT2*^ mice as described in the Methods section (Fig. S5A). Tamoxifen-induced NR1D1 overexpression in NR1D1 OVE mice was confirmed by western blot analysis (Fig. S5B). Lung tumor development in LLC1-injected NR1D1 OVE mice was dramatically reduced compared with that in LLC1-injected WT mice (Fig. 2B). These data show that NR1D1 functions as a tumor suppressor in the lung TME.

### NR1D1 acts as a tumor suppressor through the negative regulation of the NLRP3 inflammasome

**Fig. 3:**
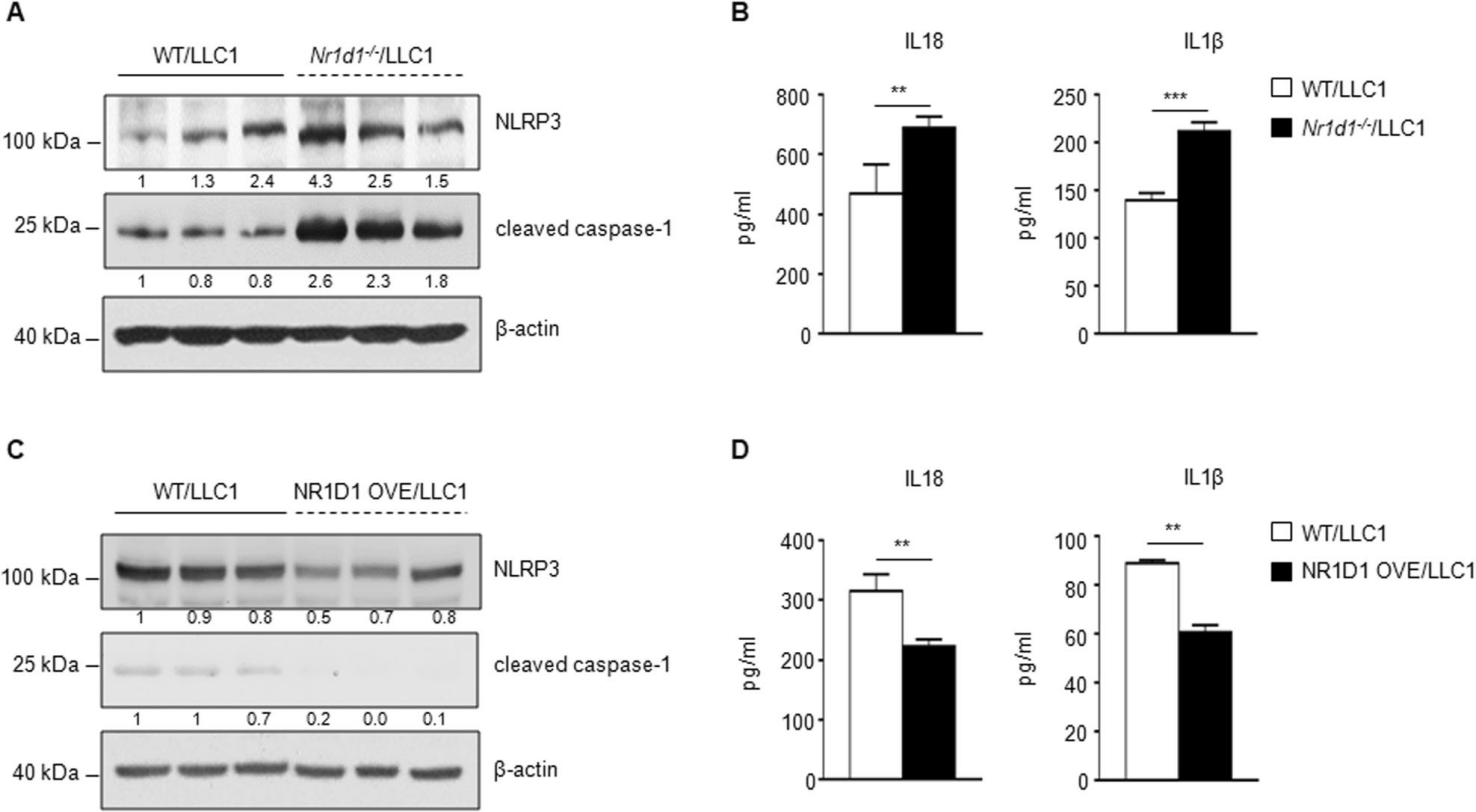
NR1D1 negatively regulates NLRP3 inflammasome activation. **A** Protein expression of NLRP3 and cleaved caspase-1 in lung tissue samples extracted from LLC1-injected WT or *Nr1d1*-null mice. Each lane indicates a lung tissue sample from a different mouse. The numbers below each blot indicate the relative expression ratio normalized to β-actin. **B** IL18 and IL1β ELISA of lung tissue samples from LLC1-injected WT (*n* = 3) and *Nr1d1*-null (*n* = 3) mice. The results are expressed as the mean ± SD. **p < 0.01; ***p < 0.001. Experiments were performed in triplicate and repeated three times. **C** Protein expression of NLRP3 and cleaved caspase-1 in lung tissue samples extracted from LLC1-injected WT and NR1D1 OVE mice. Each lane indicates a lung tissue sample from a different mouse. The numbers below each blot indicate the relative expression ratio normalized to β-actin. **D** IL18 and IL1β ELISA of lung tissue samples from LLC1-injected WT (*n* = 3) and NR1D1 OVE (*n* = 3) mice. The results are expressed as the mean ± SD. **p < 0.01. Experiments were performed in triplicate and repeated three times.

Recent studies have shown that NLRP3 inflammasome is associated with tumorigenesis of multiple types of cancer. Although the role of the NLRP3 inflammasome in tumorigenesis remains controversial, most studies have shown that the NLRP3 inflammasome is pro-tumorigenic [32]. Pourcet et al. reported that NR1D1 functions as a negative regulator of NLRP3 inflammasome activation in BMDM by blocking the transcriptions of NLRP3, IL1β, and IL18 [15]. Therefore, we hypothesized that NR1D1 suppressed lung tumor development by negatively regulating NLRP3 inflammasome activation in the TME. First, we investigated whether NLRP3 inflammasome was activated in the tumor-bearing lungs of NR1D1-deficient mice. NLRP3 protein expression was higher in NR1D1-deficient mice than in the WT mice (Fig. 3A). The protein levels of the cleaved form of caspase-1, mature IL1β, and mature IL18, which are markers of NLRP3 inflammasome activation, were also increased by NR1D1 deficiency (Fig. 3A, B). Conversely, the protein levels of IL18 and IL1β, were decreased in the lungs of NR1D1 OVE mice (Fig. 3C, D).

To ascertain NR1D1 deficiency-mediated NLRP3 inflammasome activation within TME, we examined the markers of NLRP3 inflammasome activation in alveolar macrophages (AM) and normal lung tissues without tumors. Although macrophages are a major source of IL1β, several reports have demonstrated that NLRP3 inflammasome activation and IL1β secretion also occur in endothelial cells and various types of epithelial cells, such as lung epithelial cells [33–38]. Consistent with the findings obtained from tumor-bearing lung tissues, NR1D1 deficiency led to increased mRNA expression of *Nlrp3*, *Il1β*, and *Il18* in AM, as well as increased protein expression of NLRP3 and cleaved caspase-1 in both AM and normal lung tissues (Fig. S6). These results suggest that NR1D1 in mice can negatively regulate NLRP3 inflammasome activation not only in macrophages but also in other cell types, such as lung epithelial cells, within the lung tumor microenvironment.

**Fig. 4:**
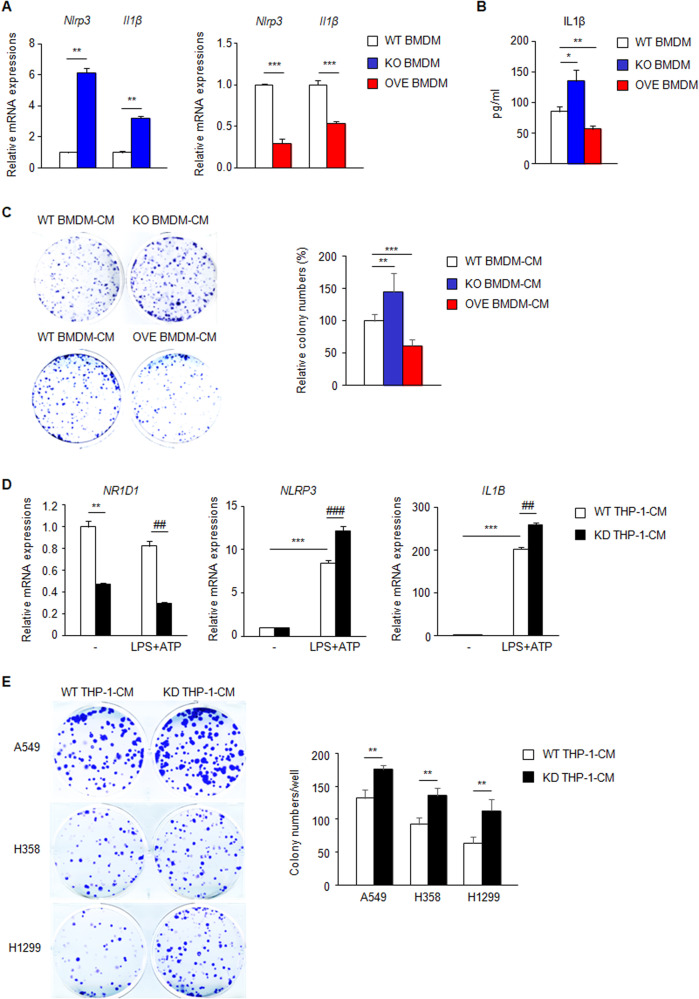
NR1D1 deficiency-mediated NLRP3 inflammasome activation in macrophages promotes the proliferation of lung cancer cells. **A** Relative mRNA expression of *Nlrp3* and *IL1β* in BMDM isolated from WT, *Nr1d1*^*−/−*^, and OVE mice. The results are expressed as the mean ± SD. **p < 0.01; ***p < 0.001. Experiments were performed in triplicate and repeated three times. **B** IL1β ELISA of WT, *Nr1d1* KO, and OVE BMDM-CM. The results are expressed as the mean ± SD. *p < 0.05; **p < 0.01. Experiments were performed in triplicate and repeated three times. **C** The upper panel shows representative images from clonogenic assay for WT, *Nr1d1* KO, or OVE BMDM-CM-treated LLC1. The lower panel shows the relative colony numbers compared to WT BMDM-CM-treated LLC1. The results are expressed as the mean ± SD. **p < 0.01; ***p < 0.001. Experiments were performed in triplicate and repeated three times. **D** Relative mRNA expression of *NR1D1*, *NLRP3*, and *IL1B* in differentiated WT or *NR1D1* KD THP-1 cells. The results are expressed as the mean ± SD. ** and ^##^*p* < 0.01; *** and ^###^*p* < 0.001. Experiments were performed in triplicate and repeated three times. **E** The left panel shows representative images from clonogenic assay for WT or *NR1D1* KD THP-1-CM-treated NSCLC cells. The right panel shows the colony numbers per well. The results are expressed as the mean ± SD. **p < 0.01. Experiments were performed in triplicate and repeated three times.

It is well established that BMDM infiltrate the tumor region and polarize with tumor-associated macrophages (TAM), a major component of the TME, thereby regulating tumor proliferation, metastasis, and angiogenesis [39]. Therefore, we examined whether NR1D1 regulates the macrophage-mediated proliferation of cancer cells. BMDM were isolated from *Nr1d1*^*−/*−^ or NR1D1 OVE mice (*Nr1d1* KO or NR1D1 OVE BMDM, respectively) and then treated with LPS for priming and ATP to activate the NLRP3 inflammasome. The expression of *Nlrp3* mRNA was increased in *Nr1d1* KO BMDM and decreased in NR1D1 OVE BMDM compared to that in WT BMDM (Fig. 4A). Also, *IL1β* mRNA and protein levels showed a similar pattern to that of NLRP3 (Fig. 4A, B). To analyze macrophage-mediated tumor growth, LLC1 cells were treated with CM obtained from WT, *Nr1d1* KO, or NR1D1 OVE BMDM. As expected, LLC1 proliferation increased upon treatment with *Nr1d1* KO BMDM-CM and decreased upon treatment with NR1D1 OVE BMDM-CM compared to that upon treatment with WT BMDM-CM (Fig. 4C). To confirm whether these results are reproduced in human cells, we induced the knockdown of *NR1D1* (*NR1D1* KD) in human monocyte THP-1 cells. We then differentiated THP-1 cells into macrophage by PMA treatment. Upon treatment with LPS and ATP, *NR1D1* KD increased the mRNA expression of *NLRP3* and *IL1B* in differentiated THP-1 cells (Fig. 4D). Consistent with the results for CM from *Nr1d1* KO BMDM, treatment with CM from *NR1D1*-knockdown THP-1 macrophages increased the proliferation of NSCLC cells (A549, H358 and H1299) (Fig. 4E).

Furthermore, we examined whether IL1β and IL18, which are the downstream mediators of the NLRP3 inflammasome, can promote the proliferation of lung cancer cells. Previous studies have reported that IL1β can enhance the proliferation of A549 and LLC1 cells [40]. Additionally, it has been suggested that IL18 may promote lung cancer cell proliferation and contribute to lung cancer progression [41]. Consistent with these reports, our findings demonstrated that IL1β and IL18 indeed stimulate the proliferation of human NSCLC cells and LLC1 (Fig. S7).

**Fig. 5:**
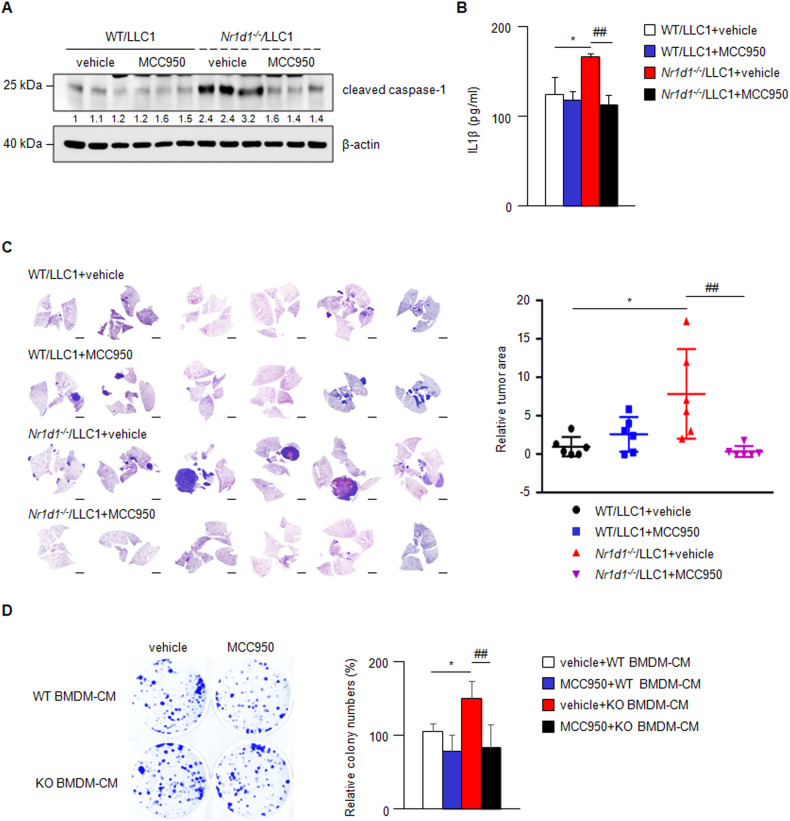
Blockade of NLRP3 inflammasome suppresses NR1D1 deficiency-mediated lung cancer progression. **A**, **B** Effects of MCC950 on the expression of markers for NLRP3 inflammasome activation in BMDM samples extracted from LLC1-injected WT and *Nr1d1*^*−/−*^ mice: **A** Western blot analysis for cleaved caspase-1. Each lane indicates a BMDM sample from a different mouse. The numbers below each blot indicate the relative expression ratio normalized to β-actin. **B** ELISA for IL-1β. The result is expressed as the mean ± SD. *p < 0.05; ^##^*p* < 0.01. Experiments were performed in triplicate and repeated three times. **C** The left panel shows representative images for H&E staining for the lung of LLC1-injected WT or *Nr1d1*^*−/*−^ mice with or without MCC950 treatment. Scale bars, 2 mm. The right panel shows the relative tumor area in each group normalized to the total lung area (n = 6 in each group). The results are expressed as the mean ± SD. *p < 0.05; ^##^*p* < 0.01. **D** The left panel shows representative images from the clonogenic assay. LLC1 cells were incubated with CM from WT or NR1D1-deficient BMDM with or without MCC950 treatment. The right panel shows the relative colony numbers (% of colony number of WT BMDM-CM-treated LLC1). The results are expressed as the mean ± SD. *p < 0.05; ^##^*p* < 0.01. Experiments were performed in triplicate and repeated three times.

To examine whether NLRP3 inflammasome activation is involved in increased lung tumor development in *Nr1d1*^*−/−*^ mice, we treated mice with MCC950, a specific inhibitor of the NLRP3 inflammasome, in orthotopic LLC1 lung cancer models. As shown in Fig. 5A, B, MCC950 treatment decreased the mRNA expression of markers for the activation of the NLRP3 inflammasome, which was increased in LLC1-injected *Nr1d1*-null mice. MCC950 treatment markedly inhibited lung tumorigenesis in LLC1-injected *Nr1d1*^−*/−*^ mice (Fig. 5C). In addition, the increased proliferation induced by *Nr1d1* KO BMDM-CM in LLC1 cells was blocked by MCC950 treatment (Fig. 5D). These results suggest that NR1D1 functions as a tumor suppressor in lung cancer by negatively regulating the NLRP3 inflammasome.

### Inhibition of NLRP3 inflammasome activation can block epithelial–mesenchymal transition induced by NR1D1 deficiency

**Fig. 6:**
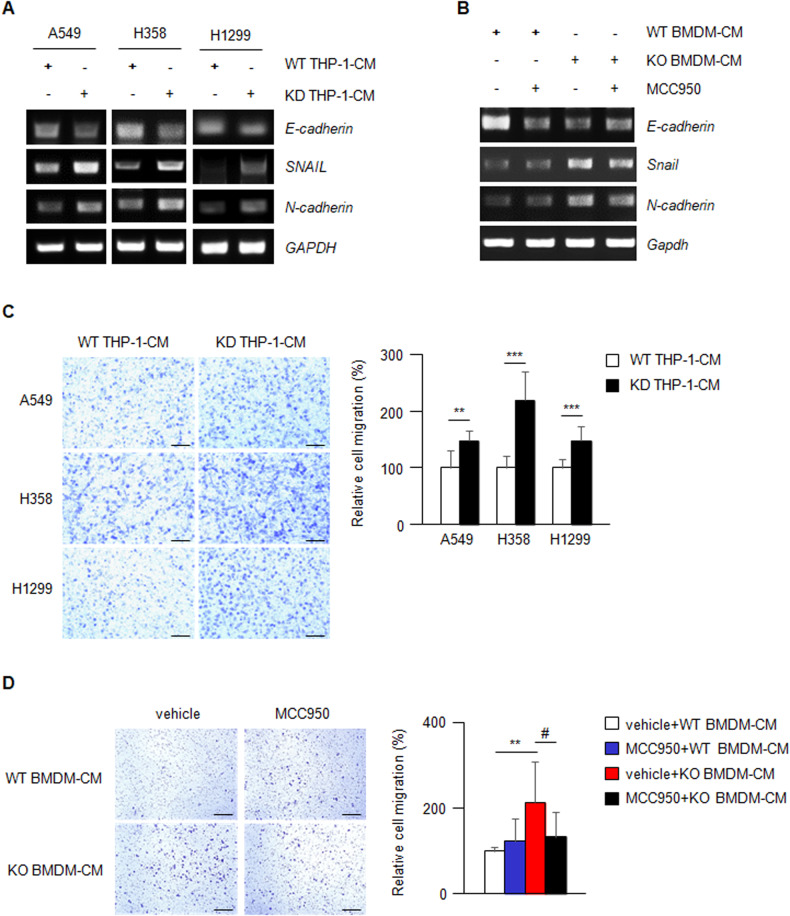
NR1D1 deficiency induces EMT in lung cancer cells in an NLRP3 inflammasome-dependent manner. **A** mRNA expression of *E-cadherin*, *N-cadherin* and *SNAIL* in WT or *NR1D1* KD THP-1-CM-treated NSCLC cells. **B** mRNA expression of *E-cadherin*, *N-cadherin*, and *Snail* in LLC1 in the presence of CM from WT or NR1D1-deficient BMDM with or without MCC950 treatment. **C** The left panel shows a representative image for the migration of WT or *NR1D1* KD THP-1-CM-treated NSCLC cells. Scale bars, 200 μm. The right panel shows the relative migratory cells (% of WT THP-1-CM treated cells). The results are expressed as the mean ± SD. **p < 0.01; ***p < 0.001. Experiments were performed in triplicate and repeated three times. **D** The left panel shows a representative image for the migration of LLC1 in the presence of CM from WT or NR1D1-deficient BMDM with or without MCC950 treatment. Scale bars, 200 μm. The right panel shows the relative migratory cells (% of WT BMDM-CM-treated LLC1). The results are expressed as the mean ± SD. **p < 0.01; ^#^*p* < 0.05. Experiments were performed in triplicate and repeated three times.

In our orthotopic lung cancer model, pleural metastasis was more prominently observed in LLC1-injected *Nr1d1*^*−/−*^ mice than in LLC1-injected WT mice (Fig. S8). Based on recent studies that NLRP3 activation promotes epithelial-mesenchymal transition (EMT) [42–44], we examined whether NR1D1 negatively regulates EMT in an NLRP3-dependent manner in vivo and in vitro. In the presence of CM obtained from *NR1D1*-knockdown THP-1 macrophages, the expression of *E-cadherin*, an epithelial marker, was reduced in the NSCLC cells (A549, H358 and H1299). Conversely, the expression of mesenchymal markers (*SNAIL* and *N-cadherin*) increased in these cells (Fig. 6A). Additionally, *Nr1d1* KO BMDM-CM reduced *E-cadherin* expression and increased *Snail* and *N-cadherin* expression in LLC1 cells (Fig. 6B). Consistent with these results, the migration of NSCLC cells was increased by the CM obtained from the *NR1D1*-knockdown THP-1 macrophages (Fig. 6C). Additionally, migration of LLC1 cells was increased by *Nr1d1* KO BMDM-CM (Fig. 6D). As expected, the increased expression of mesenchymal markers and increased migration induced by *Nr1d1* KO BMDM-CM were blocked by MCC950 in LLC1 cells, which is consistent with the results presented above (Fig. 6B, D). Taken together, these data indicate that NR1D1 deficiency in the TME positively regulates the EMT in an NLRP3 inflammasome-dependent manner.

## Discussion

Herein, we identified that NR1D1 suppresses lung tumorigenesis by negatively regulating NLRP3 inflammasome activation in the TME using mouse models with genetic modifications of *Nr1d1* gene. Even if several studies have been conducted on the role of NR1D1 in cancer development, most of them are reports using synthetic agonists. This study is the first to elucidate the role of NR1D1 in lung cancer development through in vivo experiments using genetically engineered mice and in vitro experiments, which will contribute to our understanding of the biological role of NR1D1.

We revealed that NR1D1 plays a tumor suppressive role in the TME during lung tumorigenesis. Similar to our results, recent studies have highlighted the role of circadian components in TME during tumorigenesis. In a breast cancer mouse model, CLOCK and BMAL1 in the TME promoted tumorigenesis and metastasis through the upregulation of WNT10A-mediated ALDH3A1 expression in cancer stem cells [45]. Additionally, in colon and breast cancer mouse models, PER2 in the TME played a critical role in tumor initiation and metastasis [46]. Additionally, PER1 and PER2 in the TME induced chemoresistance and immunosuppression in a melanoma mouse model [47]. Circadian clock regulators in the TME may play an essential role in regulating tumorigenesis, and their association with tumor growth, metastasis, and response to therapy is an attractive subject of study.

In the present study, we found that the activation of the NLRP3 inflammasome in the NR1D1-deficient TME plays a crucial role in lung cancer development and EMT. Consistent with our results, it was reported that NLRP3 inflammasome activation in macrophages facilitates lung cancer progression and migration [48]. Although we did not elucidate how NLRP3 inflammasome activation by NR1D1 deficiency in the TME promotes lung tumor development, the formation of a pro-tumorigenic microenvironment by NLRP3 inflammasome activation is expected to play a critical role. IL1β, a representative marker for NLRP3 inflammasome activation, is known to induce angiogenesis by promoting the maturation of endothelial precursor cells and tube-like structure formation together with vascular endothelial growth factor (VEGF) [49]. Additionally, it was reported that IL18 positively regulates angiogenesis by stimulating thrombospondin-1 and VEGF production [50]. Furthermore, the NLRP3 inflammasome is well known to contribute to the formation of a tumor immunosuppressive microenvironment. NLRP3 leads to the differentiation of tumor-promoting CD4^+^ T cell subsets (Th2, Th17, and regulatory T cells) [51]. Additionally, NLRP3 inflammasome stimulates the infiltration of myeloid-derived suppressor cells and TAMs [52, 53]. These previous reports suggest that NR1D1 deficiency may facilitate the formation of a pro-tumorigenic microenvironment. Finally, further investigation of NR1D1’s role in the TME will propose a therapeutic strategy to overcome lung cancer.

Previous studies, including our own, have demonstrated that NR1D1 functions as a transcriptional repressor of *Nlrp3*, pro-*IL1β*, and pro-*IL18*. The transcriptional activation of *Nlrp3*, pro-*IL1β*, and pro-*IL18* occurs during the priming stage of the NLRP3 inflammasome activation. It remains to be unclear whether NR1D1 plays a distinct role in the activation stage of NLRP3 inflammasome activation. However, it has been reported that an increase in the quantity of NLRP3 naturally leads to NLRP3 inflammasome activation [34]. Therefore, in our present study, it is expected that the increased expression of NLRP3 resulting from NR1D1 deficiency causes the NLRP3 inflammasome activation.

In this study, we attempted to verify the anti-tumor efficacy of SR9009, one of the NR1D1 agonists, in lung cancer. Although NR1D1 OE did not affect the growth of human NSCLC cells, SR9009 suppressed the growth of human NSCLC cells with or without NR1D1 expression in in vitro experiments (data not shown). This controversial result could be explained by the possibility that SR9009 has off-target effects. Consistent with our data, Dierickx et al. reported that SR9009 reduced cell viability and altered cellular metabolism in both hepatocytes and mouse ESCs lacking NR1D1/2, suggesting that SR9009 can induce potential side effects in an NR1D1/2-independent manner [54]. On the other hand, there is a possibility that the cytotoxic effect of SR9009 is the result of NR1D2 activation. NR1D2 deficiency has been reported to inhibit the proliferation of hepatocellular carcinoma and glioblastoma cancer cells, indicating that NR1D2 has oncogenic potential [55, 56]. However, as the role of NR1D2 in lung cancer is not known to date, we cannot exclude the possibility that NR1D2 acts as a tumor suppressor in lung cancer, similar to NR1D1. Indeed, several reports have shown that NR1D1 and NR1D2 have functional redundancy in regulating the circadian clock [15, 57]. Therefore, we suggest that synthetic NR1D1/2 agonists should be used, considering their side effects and target specificity. Genetic models will help to elucidate the role of each gene.

Although the tumor suppressive role of NR1D1 in lung cancer development was clearly observed in this study, the role of NR1D1 in other types of cancer remains to be elucidated. Numerous studies have shown that circadian rhythm regulators, such as PER2, CLOCK, BMAL1, CRY1, and CRY2, act as tumor suppressors in the liver, ovaries, colon, and lung [8–11]. In contrast, several reports have shown that these regulators can promote tumor development. CLOCK and BMAL1 showed oncogenic potential in acute myeloid leukemia [12]. Additionally, CRY1 and CRY2 have been reported to be closely associated with cancer development in p53 mutant mice [13]. The conflicting results of studies on the role of circadian clock genes in tumor development can be explained by the possibility that each gene acts in a tissue-specific manner. Since circadian clock-related factors are well known to have tissue-specific expression patterns and tissue-specific transcriptional regulation of target genes, their roles in tumorigenesis may be divergent from tissue to tissue [58–60]. Alternatively, the role of circadian clock genes in tumor development may be affected by environmental factors and an individual’s genetic background. It is widely known that the expression of circadian clock-related genes is affected by external factors, such as light, temperature, and metals [61]. Changes in the expression of circadian-related factors caused by genetic and environmental factors can determine the role of these genes in cancer development by influencing biological processes, such as cell proliferation and metabolism [61, 62]. Further studies on the role of NR1D1 in other cancer types and factors correlated with the role of NR1D1 are needed to understand the role of NR1D1.

In conclusion, we showed that NR1D1 deficiency in the TME promotes lung cancer development and metastatic potential through NLRP3 inflammasome activation. Our results suggest that NR1D1 acts as a tumor suppressor in lung cancer development, indicating that NLRP3 inflammasome blockade via NR1D1 activation could be a therapeutic option for lung cancer patients.

## Materials and methods

### GEPIA2 data analysis

The Gene Expression Profiling Interactive Analysis 2 (GEPIA2) database (http://gepia2.cancer-pku.cn/#index) was used to analyze a tumor/normal differential expression of *NR1D1* mRNA in various cancer types, including lung adenocarcinoma (LUAD), and the correlation between *NR1D1* expression level and overall survival rate in LUAD patients. Differential expression of *NR1D1* was analyzed based on tumor/normal samples from TCGA and the GTEx databases. The hypothetical value was set to [log_2_FC] cut-off=1, *p* value cut-off=0.01, and Match TCGA normal and GTEx data. We used the "Survival Analysis" module in GEPIA2 to obtain the overall survival rate correlated with *NR1D1* expression across different cancer types from the TCGA database. *NR1D1* expression was normalized to *GAPDH*. The cases were subdivided into high- and low-expression groups based on the quartile cut-off.

### Mouse experiments

*Nr1d1*^*−/*−^ mice were generated by zygote microinjection and the CRISPR/Cas9 system. A mixture of Cas9 protein (400 nM) and small guide RNA (sgRNA, 1200 nM) was microinjected into the pronucleus or cytoplasm of zygotes on C57BL/6 background. Target sequences in exon 2 of sgRNA were selected using the CRISPR design tool (http://crispor.tefor.net): 5’-CTA GTG GCT CCT CCC CGA GCC GG-3’ and 5’-ATG TGG GAC AAC CTT GAG TCA GG-3’. The edited indel mutations in F1 mice were confirmed by Sanger sequencing after TA cloning. Mice with 5 nucleotides (nt) deletion were used for further experiments. The Cas9 protein (EnGen Cas9 NLS) and sgRNAs were purchased from New England Biolabs (Beverly, MA, USA) and ToolGen (Seoul, Republic of Korea), respectively.

To generate conditional *Nr1d1* transgenic mice, full-length mouse *Nr1d1* complementary DNA (cDNA) replaced the tdTomato gene of the Ai9 vector [63]. Gene targeting in embryonic stem cells (ESCs) and germline transmission were performed as previously described [64]. The genotype of conditional *Nr1d1* transgenic mice was determined by PCR amplification. The primer sequences for genotyping were as follows: ROSA1 (5'-AAA GTC GCT CTG AGT TGT TAT-3'); ROSA2 (5'-GGC GGG CCA TTT ACC GTA AG-3'); ROSA3 (5'-GGA GCG GGA GAA ATG GAT ATG-3'); Nr1d1_F (5'-GAT CCC CAT CAA GCT GAT CCG G-3'); Nr1d1_R (5'-TGG ATG CTC CGG CGA AAA A-3'). The scheme for generating *Nr1d1* transgenic mice is shown in Fig. S5A. *Nr1d1* transgenic mice were crossed with *Cre*^*ERT2*^ mice (stock# 004453, Jackson Laboratory, Bar Harbor, ME, USA) to generate tamoxifen-inducible NR1D1 overexpressing mice (NR1D1 OVE). To induce NR1D1 overexpression, mice were injected intraperitoneally with 1 mg of tamoxifen (Sigma-Aldrich, St Louis, MO, USA) for five consecutive days. Three days after the final injection, mice were euthanized for further analysis or used in experiments with orthotopic cancer models.

To examine the effect of NR1D1 deficiency on *LSL-Kras*^*G12D/+*^*-*derived lung cancer development [30], *Nr1d1*^*−/−*^ mice with a 5 nt deletion were bred with *LSL-Kras*^*G12D/+*^ mice. *LSL-Kras*^*G12D/+*^ and *Nr1d1*^−/−^;*LSL-Kras*^*G12D/+*^ mice at 6-8 weeks of age were infected with 2 × 10^7^ pfu/ml of AdenoCre viruses (Vector Biolabs, Malvern, PA, USA). After eight weeks of virus inhalation, all mice were sacrificed for analysis. *LSL-Kras*^*G12D/+*^ mice were obtained from NCI Mouse Repository (Frederick, MD, USA). To generate an orthotopic lung tumor model, 2.5 × 10^5^ cells were mixed with matrigel (Corning, NY, USA) at a 1:1 ratio and orthotopically injected into the left lung of mice at 6-8 weeks of age. Two weeks after injection, all mice were sacrificed for analysis. To block NLRP3 inflammasome activation, mice were injected intraperitoneally with 200 μg of MCC950 (Sigma-Aldrich) every 2 days.

All mice used in this work were backcrossed with C57BL/6 mice for at least six generations. This study was reviewed and approved by the Institutional Animal Care and Use Committee (IACUC) of the National Cancer Center Research Institute (NCC-21-713 and NCC-21-713-001).

### Hematoxylin and eosin (H&E) staining

H&E staining was performed as previously described [64]. Briefly, the lung tissues were isolated from mice and fixed overnight in 10% neutral buffered formalin. After paraffin embedding, the specimens were sectioned at 4 μm and stained with H&E according to the manufacturer’s protocol (Sigma-Aldrich). The stained samples were scanned using MoticEasyScan One (Motic Asia, Kowloon, Hong Kong). The tumor area and number were measured using ImageJ software.

### Cell culture

A549, H358, and H1299 cells were obtained from Dr. Mi Kyung Park (Dongguk University, Seoul, Republic of Korea). Mouse Lewis lung carcinoma (LLC1) cells and L929 cells were purchased from American Type Cell Collection (ATCC, Rockville, MD, USA). THP-1 cells were obtained from Dr. Beom-Kyu Choi (National Cancer Center, Gyeonggi, Republic of Korea). All cells were maintained in Dulbecco’s modified Eagle’s medium (DMEM) supplemented with 10% fetal bovine serum (FBS) and 5000 units/ml penicillin/streptomycin in a humidified incubator with 5% CO_2_ at 37 °C and subcultured at least three times before usage in any experiments.

### Western blot analysis

Western blot analysis was performed as previously described [64]. Briefly, equal amounts of protein were separated by SDS-PAGE and transferred to a nictocellulose membrane (Bio-Rad, Hercules, CA, USA). After blocking with 5% bovine serum albumin, membranes were incubated with primary antibodies at 4 °C overnight, followed by incubation with horseradish peroxidase (HRP)-conjugated secondary antibodies. Blots were detected using an ECL chemiluminescence detection system (Abfrontier, Seoul, Republic of Korea). Antibodies for western blotting were purchased from the following sources: Anti-NR1D1 (14506-1-AP) and anti-NLRP3 (NBP-12446) were purchased from Proteintech (Chicago, IL, USA) and Novus Biologicals (Littleton, CO, USA), respectively. Anti-caspase-1 (sc-398715) and anti-β-actin (sc-47778) were purchased from Santa Cruz Biotechnology (Dallas, TX, USA).

### Generation of *Nr1d1* KO LLC1 clones

LLC1 cells were transfected with the NR1D1 CRISPR/Cas9 plasmid (Santa Cruz Biotechnology) using the jetPEI® DNA transfection reagent (Polyplus-transfection, Ilkirch, France) according to the manufacturer’s instruction. After clonal selection, the clones were screened for *Nr1d1* knockout (KO) by western blot analysis.

### Enzyme–linked immunosorbent assay (ELISA)

ELISA for IL18 and IL1β was performed using the Mouse IL18 ELISA Kit (Invitrogen, Carlsbad, CA, USA) and Mouse IL1β ELISA Kit (Invitrogen), respectively, according to the manufacturer’s protocol. 50 μl tissue lysates or cell culture supernatants and 50 μl biotin-conjugates were added to the primary antibody-coated wells. After incubation for 2 h, streptavidin-HRP was added to each wells. After the addition of TMB substrate, the absorbance was measured at 450 nm using a microplate reader.

### Quantitative real-time PCR (qRT-PCR) and reverse transcription-PCR (RT-PCR)

Total RNA was extracted using FavorPrep^TM^ Tri-RNA reagent (FAVORGEN Biotech Corp., Taipei, Taiwan) and converted to cDNA using ReverTra Ace® qPCR RT Master Mix with gDNA Remover (TOYOBO, Osaka, Japan) according to the manufacturer’s instructions. qRT-PCR was conducted on a LgihtCycler®480 system (Roche, Mannheim, Germany) using the TB Green® Premix Ex Taq^TM^ II (Tli RNaseH Plus) (Takara, Tokyo, Japan). The expression of each mRNA was normalized to that of GAPDH. For RT-PCR, the cDNA was amplified using PCR. The PCR program was 95 °C for 5 min followed by 25–30 cycles at 95 °C for 30 s, 56 °C for 30 s, and 72 °C for 45 s. The reaction was allowed to proceed for 10 min at 72 °C. The PCR products were analyzed by electrophoresis on a 2% agarose gel. Primers used for qRT-PCR and RT-PCR are listed in Table S1.

### Clonogenic assay

For the clonogenic assay, cells were seeded in 6-well plates (500 cells/well). After 10–14 days, the cells were fixed with ice-cold methanol for 10 min and stained with a crystal violet solution (Sigma-Aldrich).

### siRNA transfection and differentiation in THP-1 cells

THP-1 cells were transfected with 50 μmol/l siRNA with the INTERFERIN® siRNA/miRNA transfection reagent (Polyplus transfection). Two days post-transfection, the cells were treated with 100 ng/ml phorbol 12-myristate 13-acetate (PMA) for 18 h. Then, the cells were treated with 100 ng/ml lipopolysaccharide (LPS) for 3 h, followed by 1 mM adenosine triphosphate (ATP) for 1 h.

### Isolation and culture of bone marrow-derived macrophages (BMDM)

Femurs and tibiae from wild-type (WT), *Nr1d1*^*−/−*^ or tamoxifen-treated NR1D1 OVE mice were harvested from both legs. Bone marrow cells were obtained by flushing with a 27-guage needle and maintained in DMEM supplemented with 10% FBS, 20% L929-conditioned medium (CM), and 5000 units/ml penicillin/streptomycin in a humidified incubator with 5% CO_2_ at 37 °C for 7 days. The bone marrow cells were then treated with 100 ng/ml LPS for 3 h and 1 mM ATP for 1 h.

### Transwell migration assay

Transwell migration assay was conducted using 24-well plates with 8 μm pore sized transwell chambers (Corning, NY, USA). After serum starvation, 5 × 10^4^ cells were added to the upper chamber and DMEM containing 30% BMDM-CM was added to the lower chamber. After 24 h, the migrated cells were fixed with ice-cold methanol and stained with a crystal violet solution (Sigma-Aldrich).

### Statistical analysis

Statistical significance between groups was assessed using the Student’s *t* test, and *p* < 0.05 was considered to be statistically significant. All data are presented as the mean ± SD. All statistical details of experiments can be found in the result and figure legends. The sample sizes can be found in the figure legends. No statistical method was used to pre-determine the sample size for mice. No randomization or blinding was used in the in vivo studies.

## Supplementary information

Supplementary information

Original western blot

## Data Availability

All data generated or analyzed during this study are available from the corresponding author upon reasonable request.
