# Supplementary material for: NR1D1 deficiency in the tumor microenvironment promotes lung tumor development by activating the NLRP3 inflammasome
Source: Cell Death Discov. 2023 Jul 31;9:278. doi: 10.1038/s41420-023-01554-3 (PMC10390518; doi:10.1038/s41420-023-01554-3)
Supplement: Supplementary file 1 — Supplementary information [file 41420_2023_1554_MOESM1_ESM.pdf]

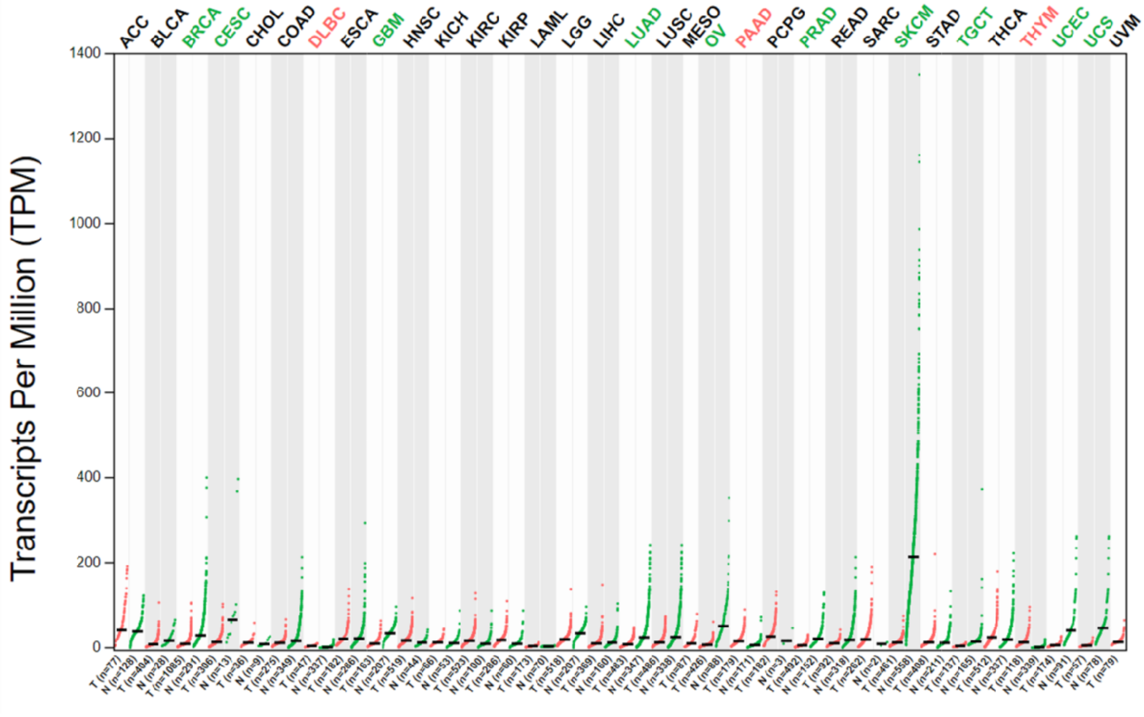
ACC
BLCA
BRCA
CESC
CHOL
COAD
DLBC
ESCA
GBM
HNSC
KICH
KIRC
KIRP
LAML
LGG
LIHC
LUAD
LUSC
MESO
OV
PAAD
PCPG
PRAD
READ
SARC
SKCM
STAD
TGCT
THCA
THYM
UCEC
UCS
UVM
Transcripts Per Million (TPM)
1400
1200
1000
800
600
400
200
0

Fig. S1: GEPIA2 data analysis for *NR1D1* expression in normal and cancer tissues.

The red and green dots indicate tumor samples and normal samples, respectively. ACC, Adrenocortical carcinoma; BLCA, Bladder urothelial carcinoma; BRCA, Breast invasive carcinoma; CESC, Cervical squamous cell carcinoma and endocervical adenocarcinoma; CHOL, Cholangio carcinoma; COAD, Colon adenocarcinoma; DLBC, Lymphoid neoplasm diffuse large B-cell lymphoma; ESCA, Esophageal carcinoma; GBM, Glioblastoma multiforme; HNSC, Head and neck squamous cell carcinoma; KICH, Kidney chromophobe; KIRC, Kidney renal clear cell carcinoma; KIRP, Kidney renal papillary cell carcinoma; LAML, Acute myeloid leukemia; LGG, Brain lower grader glioma; LIHC, Liver hepatocellular carcinoma; LUAD, Lung adenocarcinoma; LUSC, Lung squamous cell carcinoma; MESO, Mesothelioma; OV, Ovarian serous cystadenocarcinoma; PAAD, Pancreatic adenocarcinoma; PCPG, Pheochromocytoma and paraganglioma ; PRAD, Prostate adenocarcinoma; READ, Rectum adenocarcinoma; SARC, Sarcoma; SKCM, Skin cutaneous melanoma; STAD, Stomach adenocarcinoma; TGCT, Testicular germ cell tumors; THCA, Thyroid carcinoma; THYM, Thymoma; UCEC, Uterine corpus endometrial carcinoma; UCS, Uterine carcinosarcoma and UVM, Uveal melanoma.

A

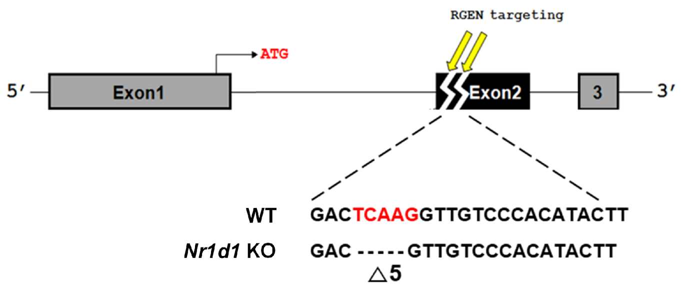

WT (615 aa ≒ 70kDa)

MTTLDSNNNTGGVITYIGSSGSSPSRTSPESLYSDSSNGSFQSLTQGCPTYFPPSPTGSLTQDPAR
SFGSAPPSLSDDSSPSSTSSSSSSSSSFYNGSPPGSLQVAMEDSSRVSPSKGTSNITKLNGMVLL
CKVCGDVASGFHYGVHACEGCKGFFRRSIQQNIQYKRCLKNENCSIVRINRNRCQQCRFKKCLSV
GMSRDAVRFGRIPKREKQRMLAEMQSAMNLANNQLSSLCPLETSPTPHPTSGSMGPSPPPAPAP
TPLVGFSQFPQQLTPPRSPSPEPTMEDVISQVARAHREIFTYAHDKLGTSPGNFNANHASGSPSAT
TPHRWESQGCPSAPNDNNLLAAQRHNEALNGLRQGPSSYPPTWPSGPTHHSCHQPNSNGHRLC
PTHVYSAPEGEAPANSLRQGNTKNVLLACPMNMYPHGRSGRTVQEIWEDFSMSFTPAVREVVEF
AKHIPGFRDLSQHDQVTLLKAGTFEVLMVRFASLFNVKDQTVMFLSRTTYSLQELGAMGMGDLLN
AMFDFSEKLNSLALTEEELGLFTAVVLVSADRSGMENSASVEQLQETLLRALRALVLKNRPSETSR
FTKLLLKLPDLRTLNNMHSEKLLSFRVDAQ **STOP**

***Nr1d*1 mutant (74 aa ≒ 8 kDa)**

MTTLDSNNNTGGVITYIGSSGSSPSRTSPESLYSDSSNGSFQSLTLSHILPTITYWLPHPGPCPLFWQCATQSQ **STOP**

B

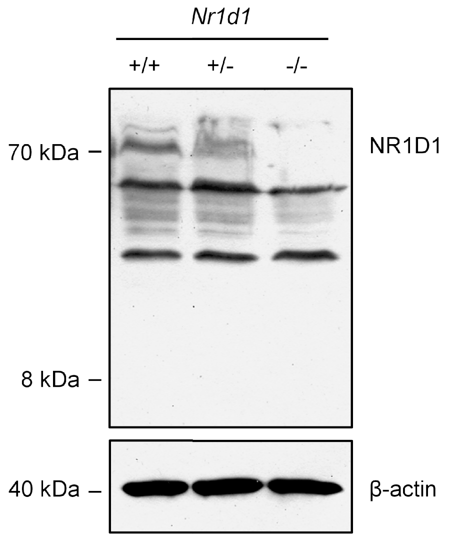

Fig. S2: Generation of *Nr1d1*$^{-/-}$ mice using the CRISPR/Cas9 system.

(A) Schematic representation for CRISPR/Cas9 targeting of *Nr1d1* exon2. The upper panel shows a partial structure of the *Nr1d1* gene on chromosome 11 and RNA-guided engineered nucleases targeted region. As a result of targeting exon 2, 5 nucleotides (nt) in exon 2 (red) were deleted. The lower panel shows amino acid sequences of *Nr1d1* WT and mutant allele. Blue, common sequence of WT and mutant allele; Red, sequence generated by 5-nt deletion-mediated frameshift.

(B) Western blot analysis with protein extract from lung tissues. +/+, WT; +/-, *Nr1d1*$^{+/-}$; -/-, *Nr1d1*$^{-/-}$ mice.

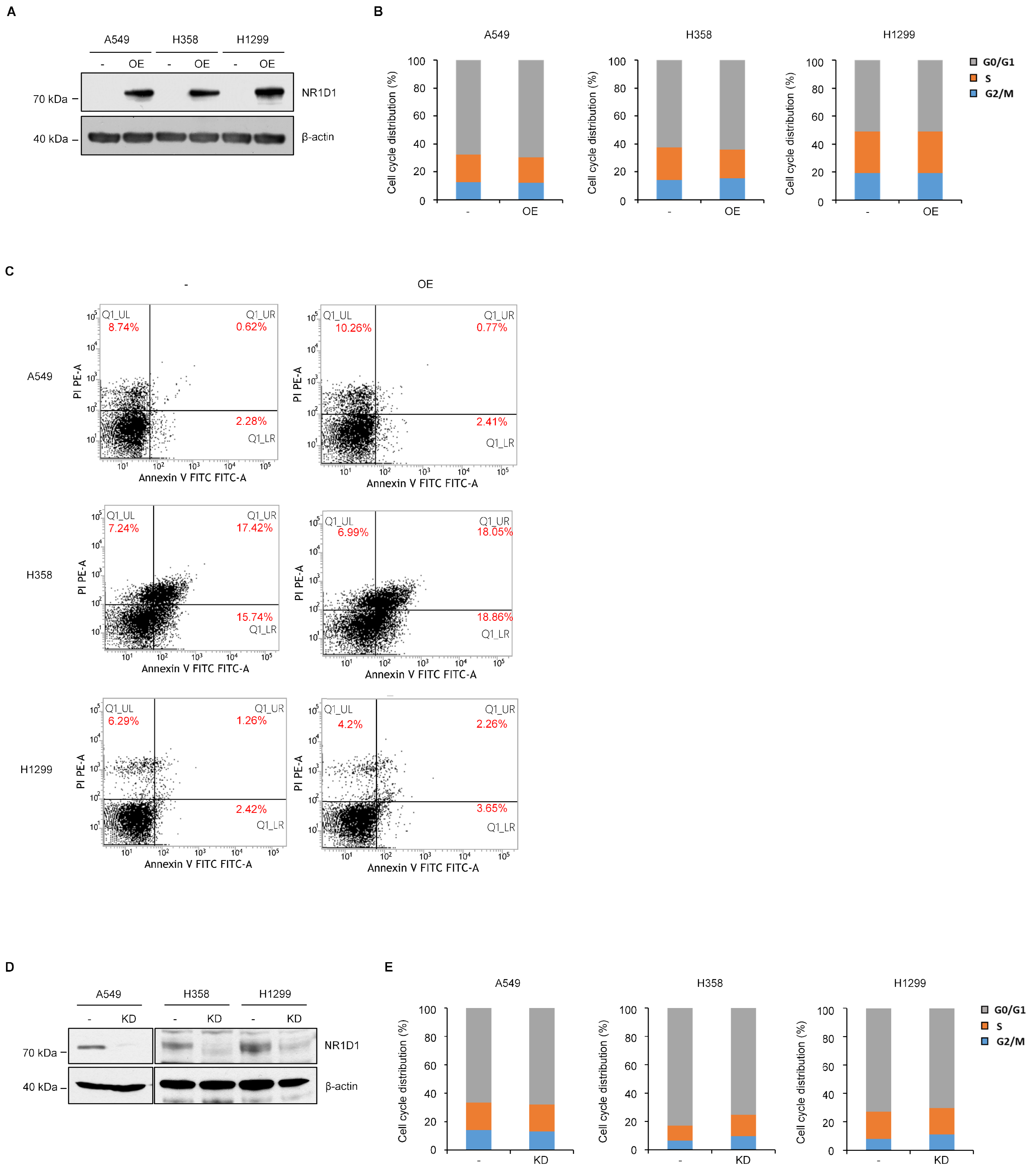
A
A549
H358
H1299
-
OE
70 kDa
40 kDa
NR1D1
β-actin
B
Cell cycle distribution (%)
G0/G1
S
G2/M
C
Q1_UL
Q1_UR
Q1_LR
PI PE-A
Annexin V FITC FITC-A
8.74%
0.62%
2.28%
10.26%
0.77%
2.41%
7.24%
17.42%
15.74%
6.99%
18.05%
18.86%
6.29%
1.26%
2.42%
4.2%
2.26%
3.65%
D
KD
E

Fig. S3: *NR1D1* OE or KD in NSCLC cells does not affect cell cycle distribution and cell apoptosis.

(A) Western blot analysis for NR1D1-overexpressing NSCLC cells.

(B) Effect of *NR1D1* OE on cell cycle distribution of NSCLC cells.

(C) Cell apoptosis analysis for *NR1D1* WT and OE NSCLC cells.

(D) Western blot analysis for *NR1D1*-knockdown NSCLC cells.

(E) Effect of *NR1D1* KD on cell cycle distribution of NSCLC cells.

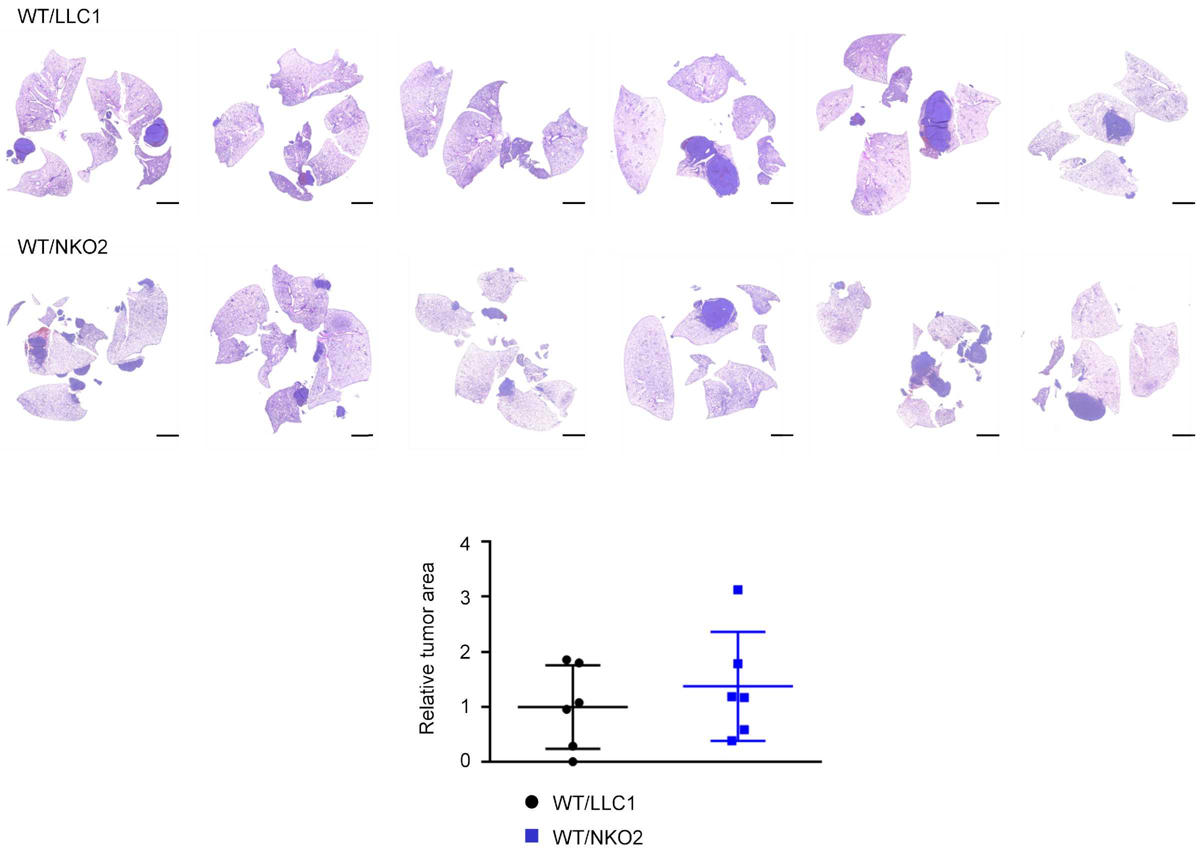

Fig. S4: NR1D1 deficiency in cancer cells does not affect lung cancer development.

The upper panel shows images from H&E staining for the lung of LL1 or NKO2-injected WT mice. Scale bars, 2 mm. The lower panel indicates the relative tumor area (normalized to total lung) between groups (n=6 in each group).

A

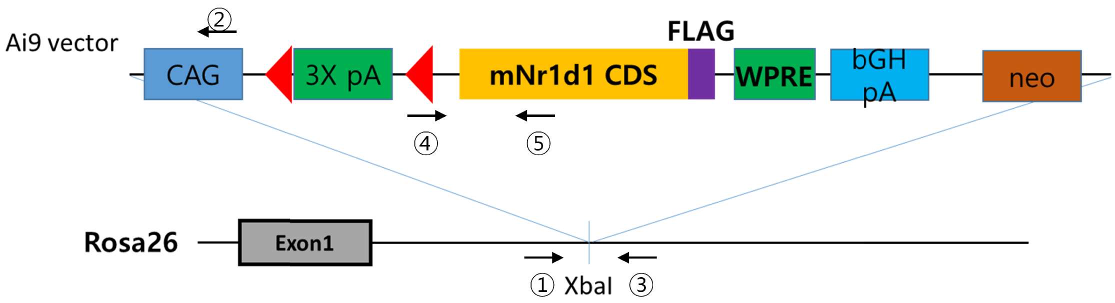

B

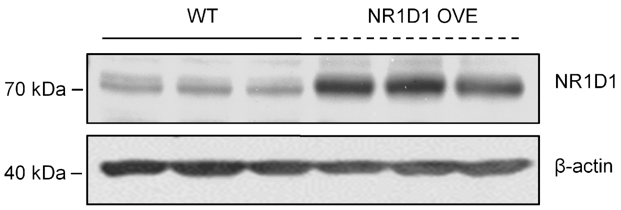

Fig. S5: Generation of *Nr1d1* transgenic mice.

(A) Schematic representation for the targeting vector and targeted locus. The transgenic mice were generated with modified Ai9 vector as previously described [1]. Black arrows indicate position of primers for genotyping (①, ROSA1; ②, ROSA2; ③, ROSA3; ④, Nr1d1_F; ⑤, Nr1d1_R).

(B) Western blot analysis for NR1D1 expression in lungs of WT mice and tamoxifen-treated *Nr1d1* transgenic mice (NR1D1 OVE).

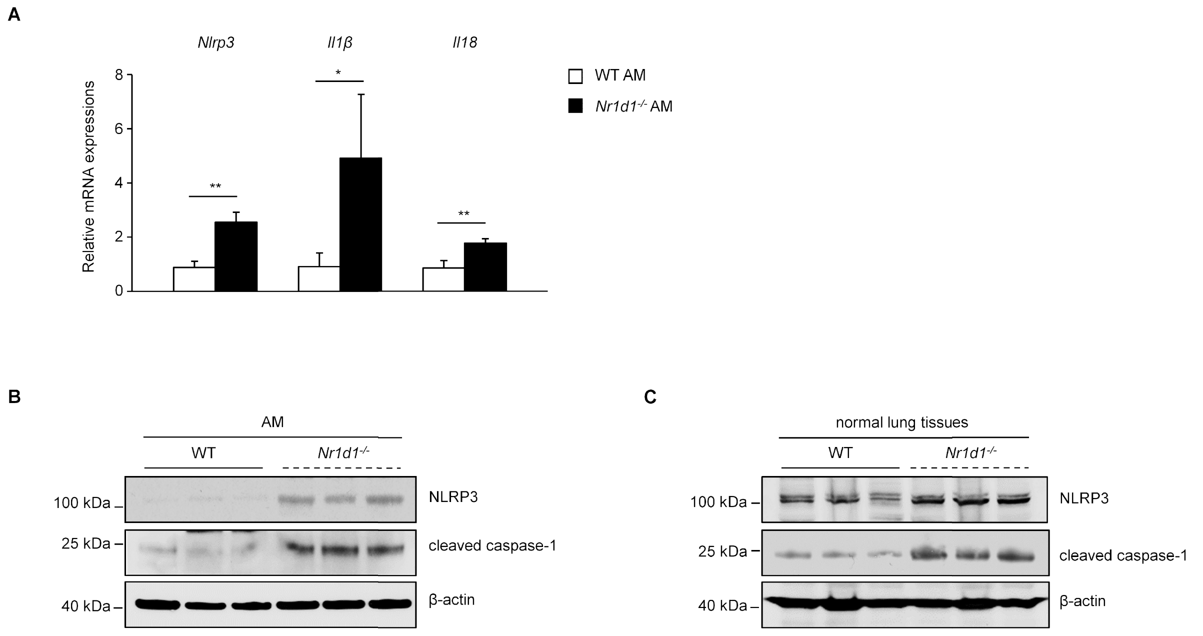

Fig. S6: NR1D1 deficiency-mediated NLRP3 inflammasome activation in alveolar macrophages (AM) and normal lung tissues.

(A) Relative mRNA expression of *Nlrp3*, *IL1β* and *IL18* in AM isolated from WT and *Nr1d1*$^{-/-}$ mice. The results are expressed as the mean ± SD. * $p < 0.05$; ** $p < 0.01$.

(B) Protein expression of NLRP3 and cleaved caspase-1 in AM samples extracted from WT or *Nr1d1*-null mice. Each lane indicates a protein sample extracted from the combined AM of two mice.

(C) Protein expression of NLRP3 and cleaved caspase-1 in normal lung tissues samples extracted from WT or *Nr1d1*-null mice. Each lane indicates a lung tissue sample from a different mouse.

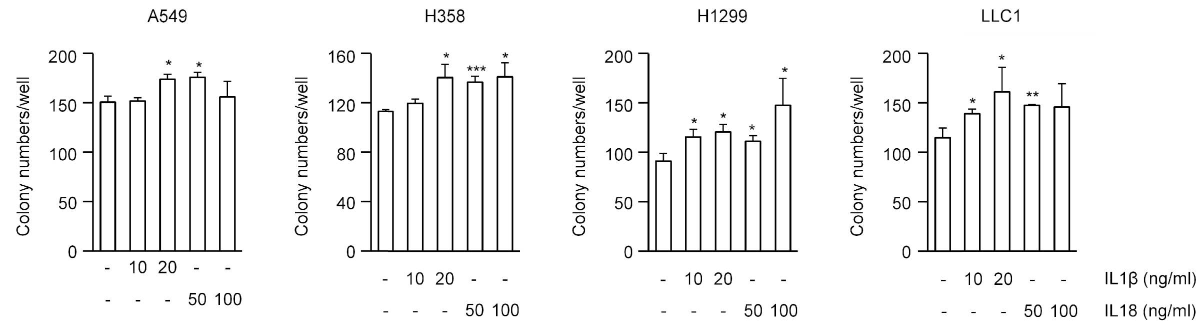

Fig. S7: Effects of IL1β and IL18 on the lung cancer cells proliferation

The graphs show the colony numbers per well. The results are expressed as the mean± SD. * $p < 0.05$; ** $p < 0.01$; *** $p < 0.001$. Experiments were performed in triplicate and repeated three times.

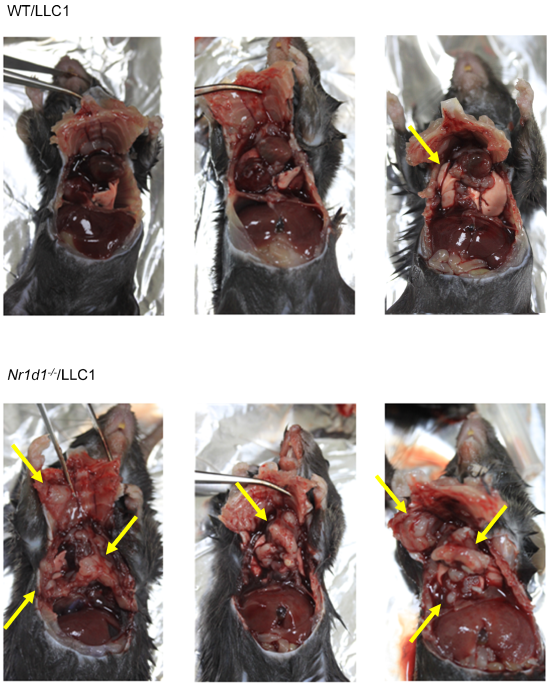

	total mice	mice with pleural metastasis
WT/LLC1	5	2
Nr1d1$^{-/-}$/LLC1	5	5

Fig. S8: Increased pleural metastasis in LLC-injected *Nr1d1*$^{-/-}$ mice.

The left panel shows representative images for pleural metastasis in LLC1-injected WT and *Nr1d1*$^{-/-}$ mice. The yellow arrows indicate pleural metastasis. The right panel shows the number of mice harboring pleural metastasis in LLC1-injected WT and *Nr1d1*$^{-/-}$ mice.

SUPPLEMENTARY METHODS

Cloning of human NR1D1 expression vector and transient transfection

NR1D1/pcDNA3.1 was constructed by inserting human *NR1D1* cDNA into the pcDNA3.1 vector. For overexpression of NR1D1, the mixture of NR1D1/pcDNA3.1 and jetPEI® DNA transfection reagent (Polyplus-transfection, Ilkirch, France) was prepared according to the instructions of the manufacturer's manual. And then, $2X10^5$ cells were incubated with the mixture for 48 h.

Cell cycle analysis

Cell cycle analysis was performed as previously described [2]. Briefly, $2X10^5$ cells were transfected with NR1D1/pcDNA3.1. After 48 h, cells were harvested by trypsinization. After fixation with ice-cold 70% ethanol at 4 °C, cells were treated with 0.5mg/ml RNase A (Sigma-Aldrich) for 10 min at 37 °C. After staining with 50 ㎍/㎖ propidium iodide (PI, Sigma-Aldrich), cell cycle distribution was analyzed from $1X10^4$ cells using flow cytometry (BD Biosciences, San Diego, CA, USA).

Cell apoptosis analysis

Apoptosis analysis was performed using FITC Annexin V Apoptosis Detection Kit according to the manufacturer's manual (BD biosciences). Briefly, $1X10^5$ cells were incubated with Annexin V and PI for 15 min at room temperature and then analyzed by flow cytometry.

Alveolar macrophages (AM) isolation

AM were isolated using the following procedure, as previously described [3, 4]. AM were isolated from 6-8-week-old mice. After tracheal intubation by inserting a catheter, AM were obtained by performing bronchoalveolar lavage (BAL). The cell pellet obtained after centrifugation for 10 min at 400 g was used for subsequent analysis.

Clonogenic assay

For the clonogenic assay, cells were seeded in 6 well plates (500 cells/well) and then treated with IL1β and IL18 (R&D systems, Inc., MN, USA). After 10-14 days, the cells were fixed with ice-cold methanol for 10 min and stained with a crystal violet solution (Sigma-Aldrich).

Table S1. **List of primer sequences for qRT-PCR and RT-PCR**

Gene	Primer sequence	
mouse *Nlrp3*	sense<br>antisense	5’-CCCTTGGAGACACAGGACTC-3’<br>5’-GAGGCTGCAGTTGTCTAATTCC-3’
mouse *Il18*	sense<br>antisense	5’-TCAAAGTGCCAGTGAACCCC-3’<br>5’-GGTCACAGCCAGTCCTCTTAC-3’
mouse *Il1β*	sense<br>antisense	5’-AATGCCACCTTTTGACAGTGATG-3’<br>5’-AGCTTCTCCACAGCCACAAT-3’
mouse *E-cadherin*	sense<br>antisense	5’-GAAGGCTTGAGCACA ACAGC-3’<br>5’-CCGGGCATTGACCTCATTCT-3’
mouse *N-cadherin*	sense<br>antisense	5’-GGAGCCTATGAAGGAACCACA-3’<br>5’-GCAAGTTGATTGGCGGGATG-3’
mouse *Snail*	sense<br>antisense	5’-TCTGCACGACCTGTGGAAAG-3’<br>5’-GTTGGAGCGGTCAGCAAAAG-3’
mouse *Gapdh*	sense<br>antisense	5’-TGTCGTGGAGTCTACTGGTGTC-3’<br>5’-GCTAAGCAGTTGGTGGTGCAGG-3’
human *NR1D1*	sense<br>antisense	5’-GTGACAACTCCAATGGCAGC-3’<br>5’-CTGGGTGGAATGCTCCCAAA3’
human *NLRP3*	sense<br>antisense	5’-GAGGAAAAGGAAGGCCGACA3’<br>5’-CCCGGCAAAAACTGGAAGTG-3’
human *IL1B*	sense<br>antisense	5’-TCGCCAGTGAAATGATGGCT-3’<br>5’-AGGTCCTGGAAGGAGCACTT-3’
human *E-cadherin*	sense<br>antisense	5’-GTCATCCAACGGGAATGCA-3’<br>5’-TGATCGGTTACCGTGATCAAAA-3’
human *SNAIL*	sense<br>antisense	5’-TTCAACTGCAAATACTGCAACAAG-3’<br>5’-CGTGTGGCTTCGGATGTG-3’
human *N-cadherin*	sense<br>antisense	5’-TGGAGAACCCCATTGACATT-3’<br>5’-TGATCCCTCAGGAACTGTCC-3’
human *GAPDH*	sense<br>antisense	5’-CTCCAAAATCAAGTGGGGCG-3’<br>5’-GGGCAGAGATGATGACCCTT-3’

SUPPLEMENTARY REFERENCES

1. Madisen L, Zwingman TA, Sunkin SM, Oh SW, Zariwala HA, Gu H, et al. A robust and high-throughput Cre reporting and characterization system for the whole mouse brain. Nat Neurosci. 2010;13:133-140.

2. Kim SM, Jeon Y, Kim D, Jang H, Bae JS, Park MK, et al. AIMP3 depletion causes genome instability and loss of stemness in mouse embryonic stem cells. Cell Death Dis. 2018;9:972-985.

3. Krogsaeter EK, Spix B, Butz E, Rosato AS, Grimm C. Isolation and cell culture of primary alveolar macrophages. Bio-protocol Preprint. 2020.

4. Plesch E, Chen C, Butz E, Rosato AS, Krogsaeter EK, Yinan H, et al. Selective agonist of TRPML2 reveals direct role in chemokine release from innate immune cells. eLIFE. 2018;7:e39720.
