# Supplementary figures and images for: NR1D1 deficiency in the tumor microenvironment promotes lung tumor development by activating the NLRP3 inflammasome
Source: Cell Death Discov. 2023 Jul 31;9:278. doi: 10.1038/s41420-023-01554-3 (PMC10390518; doi:10.1038/s41420-023-01554-3)

Fig. 2A

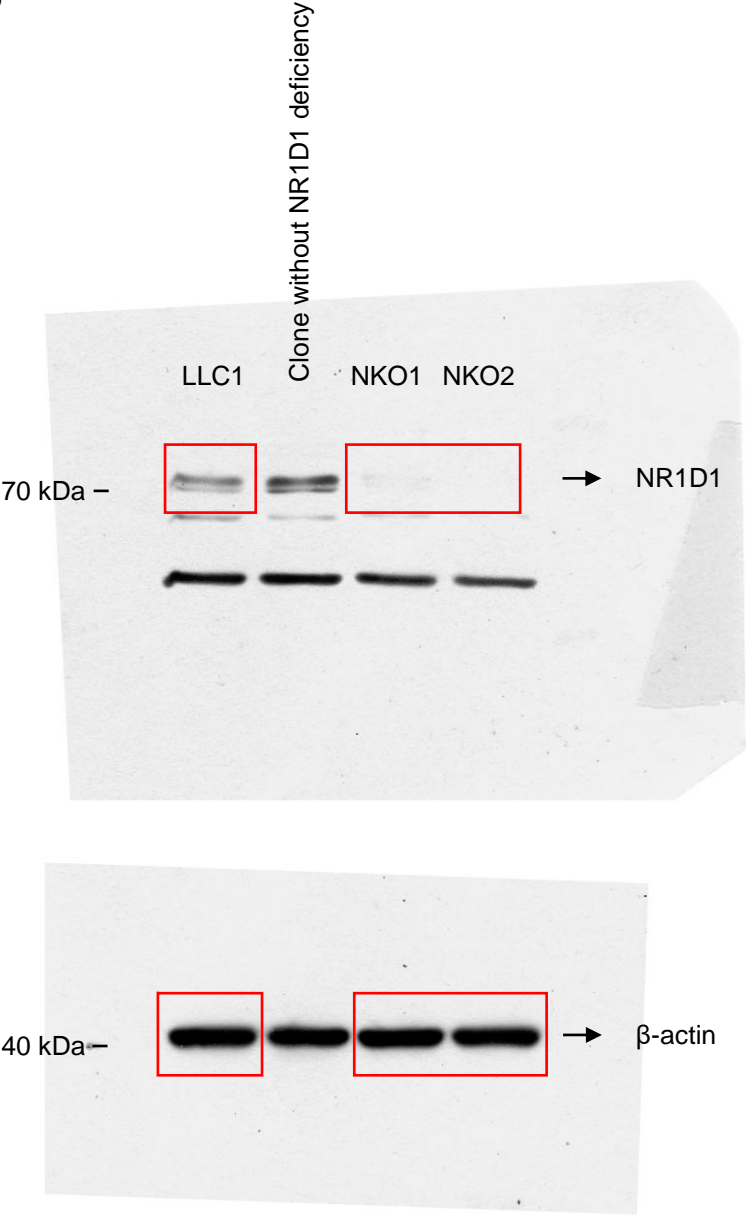

Fig. 3A

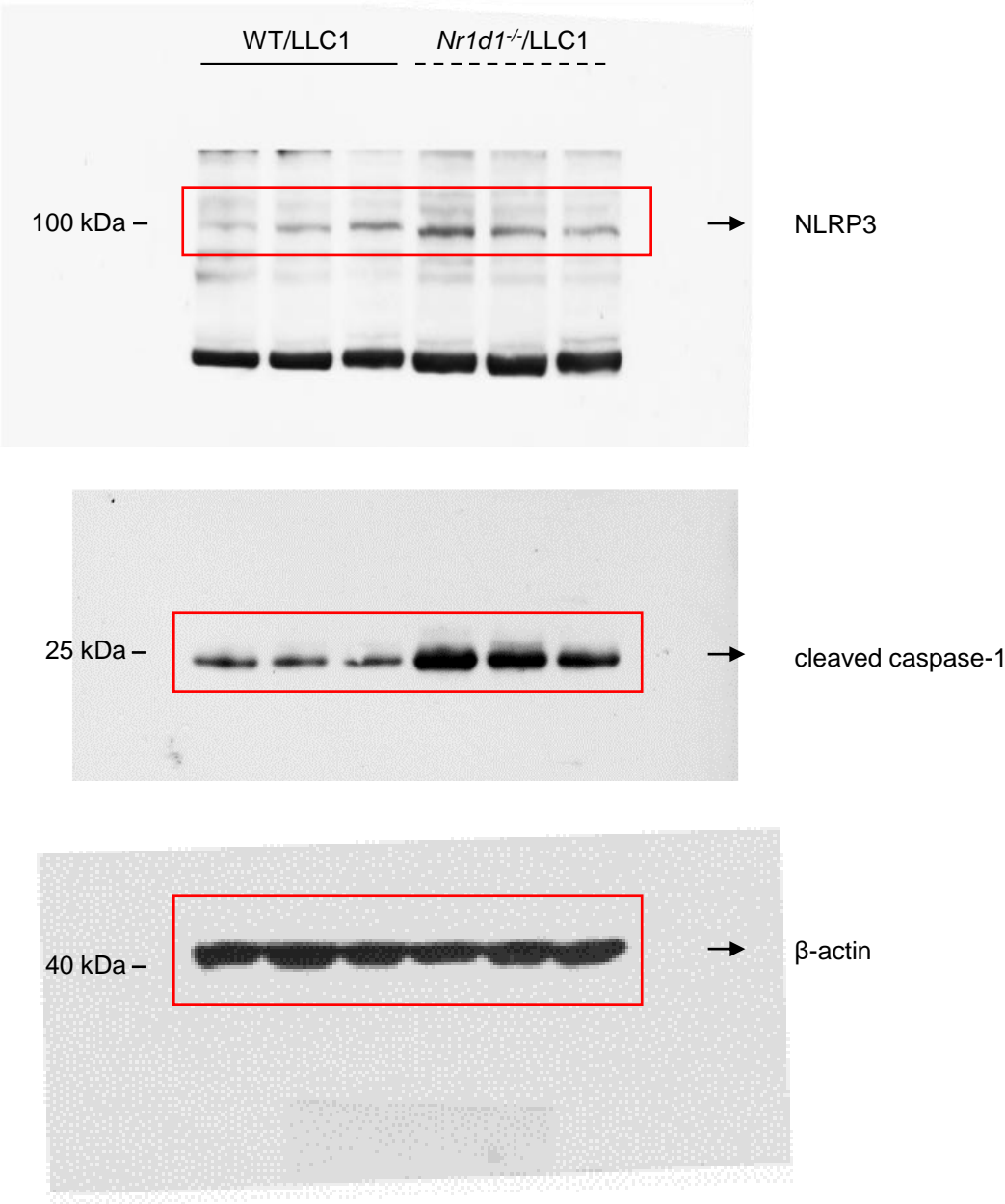

Fig. 3C

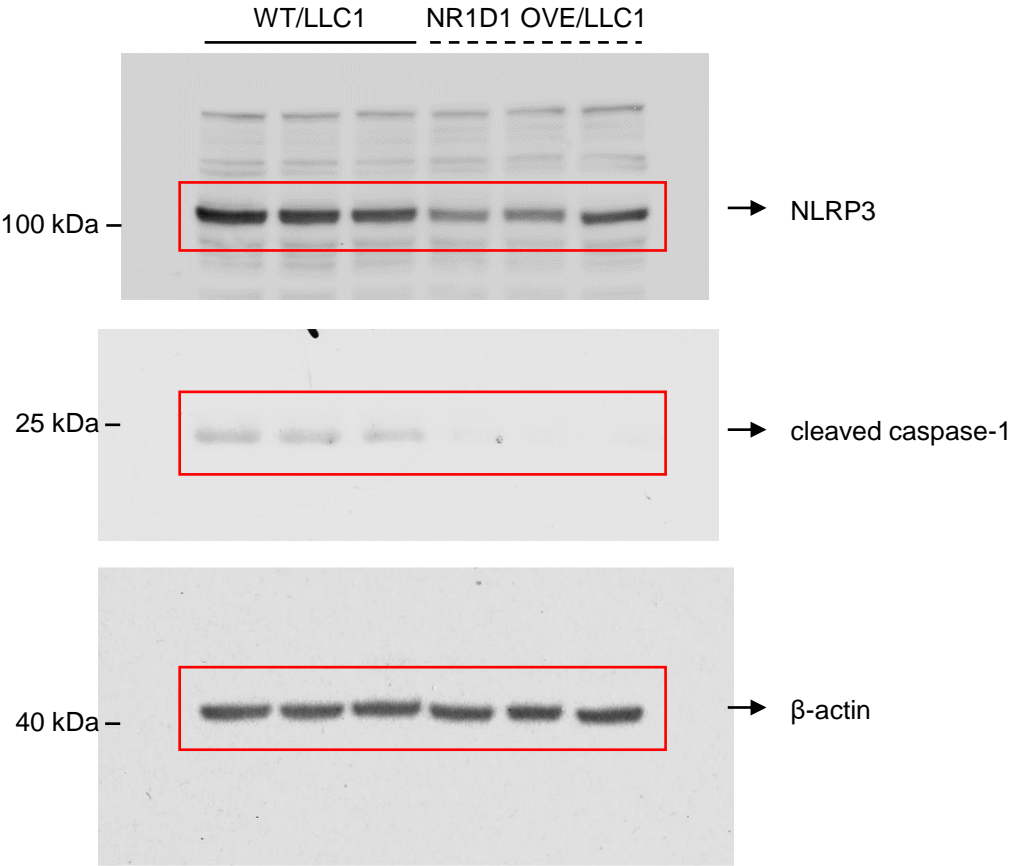

Fig. 5A

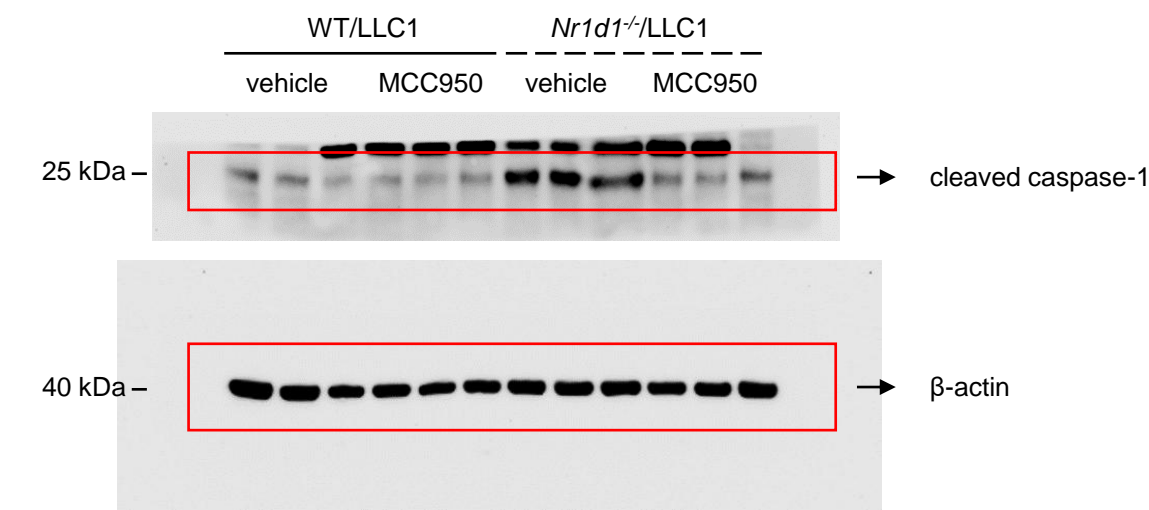

Fig. S2B

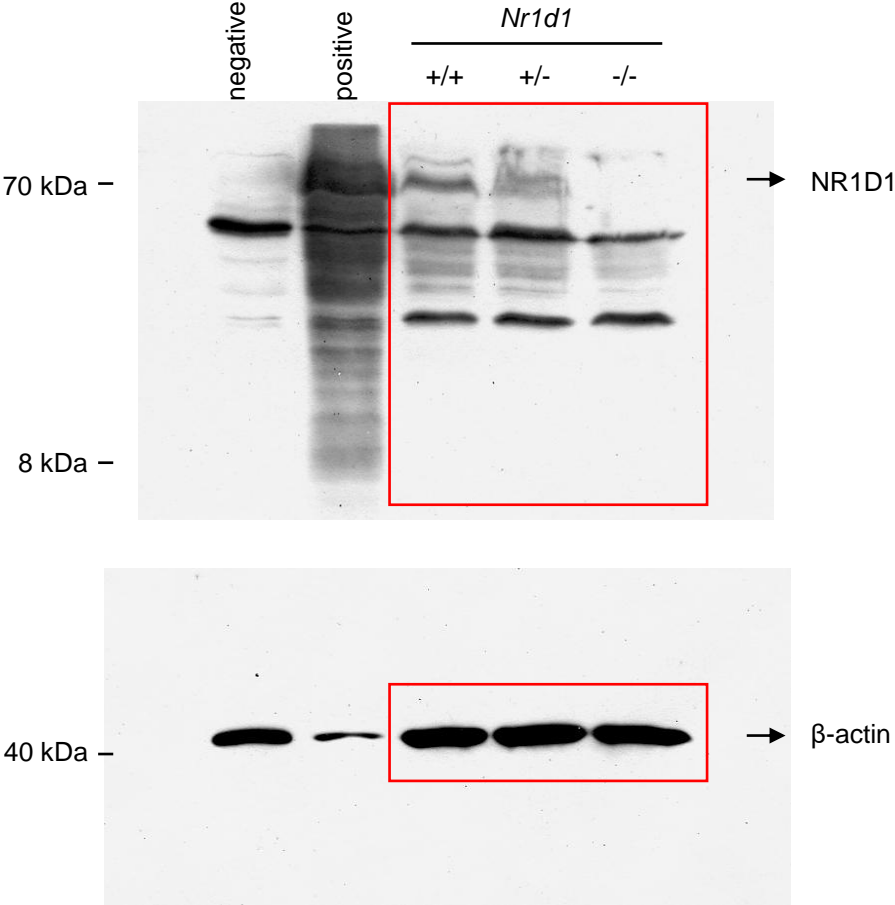

Fig. S3A

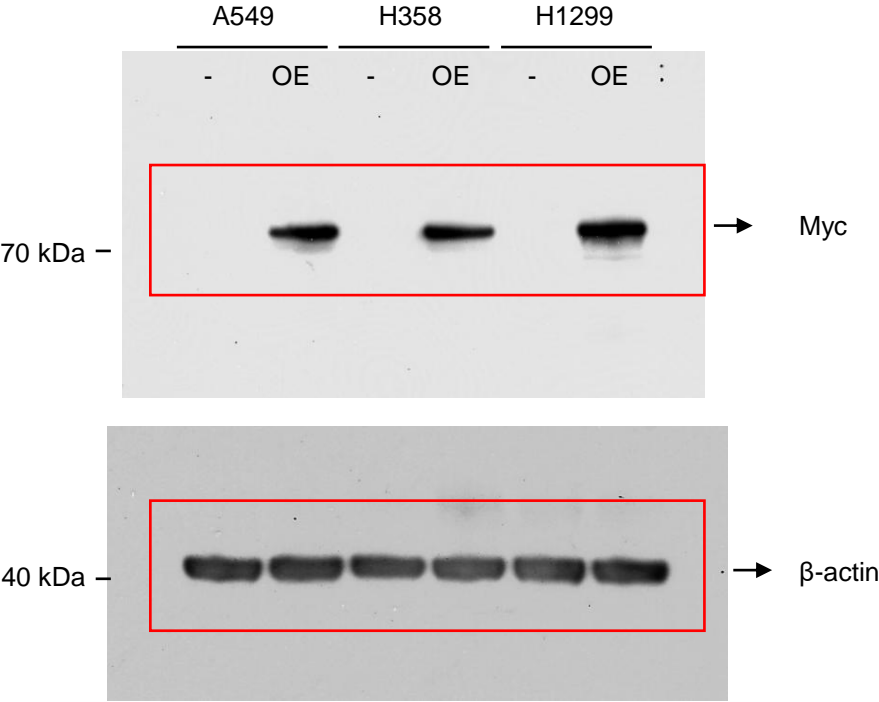

Fig. S3D

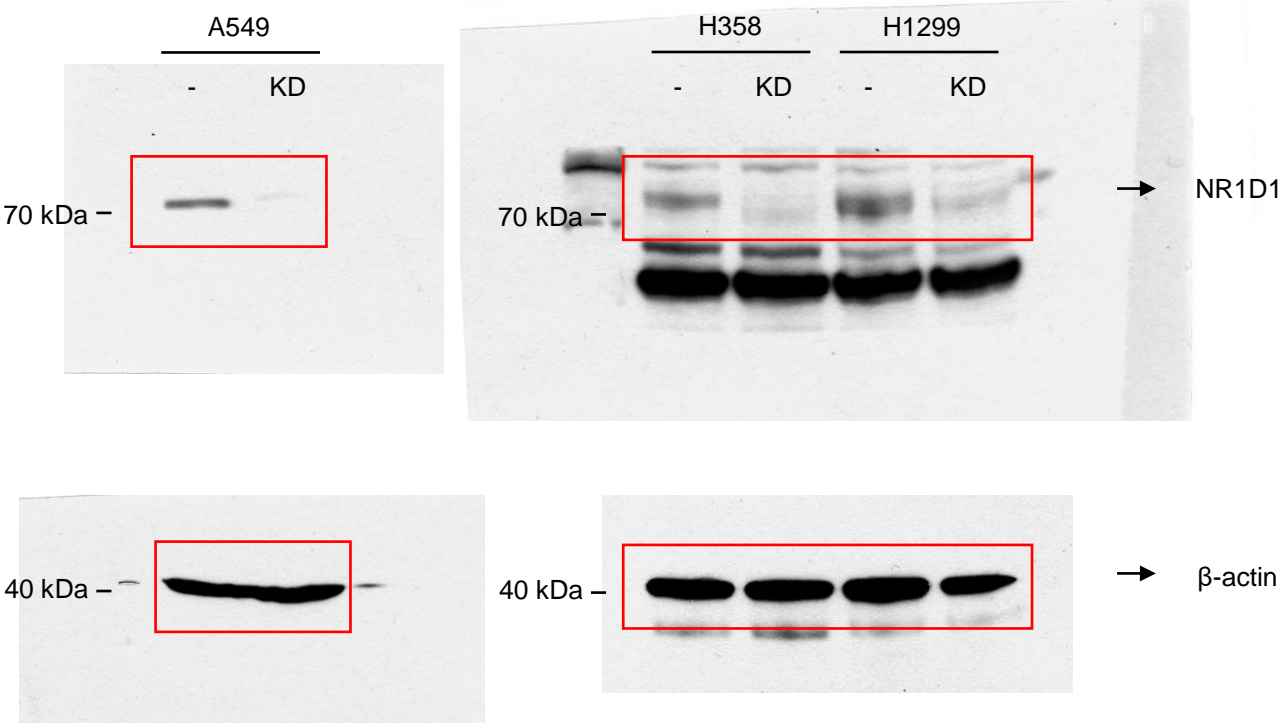

Fig. S5B

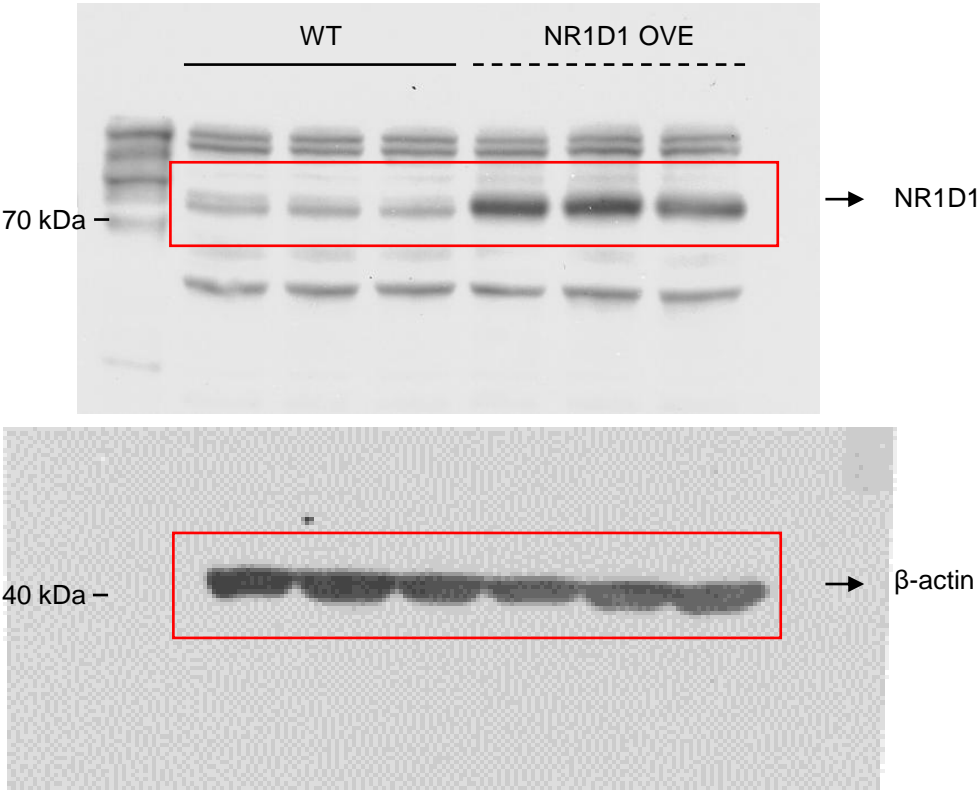

Fig. S6B

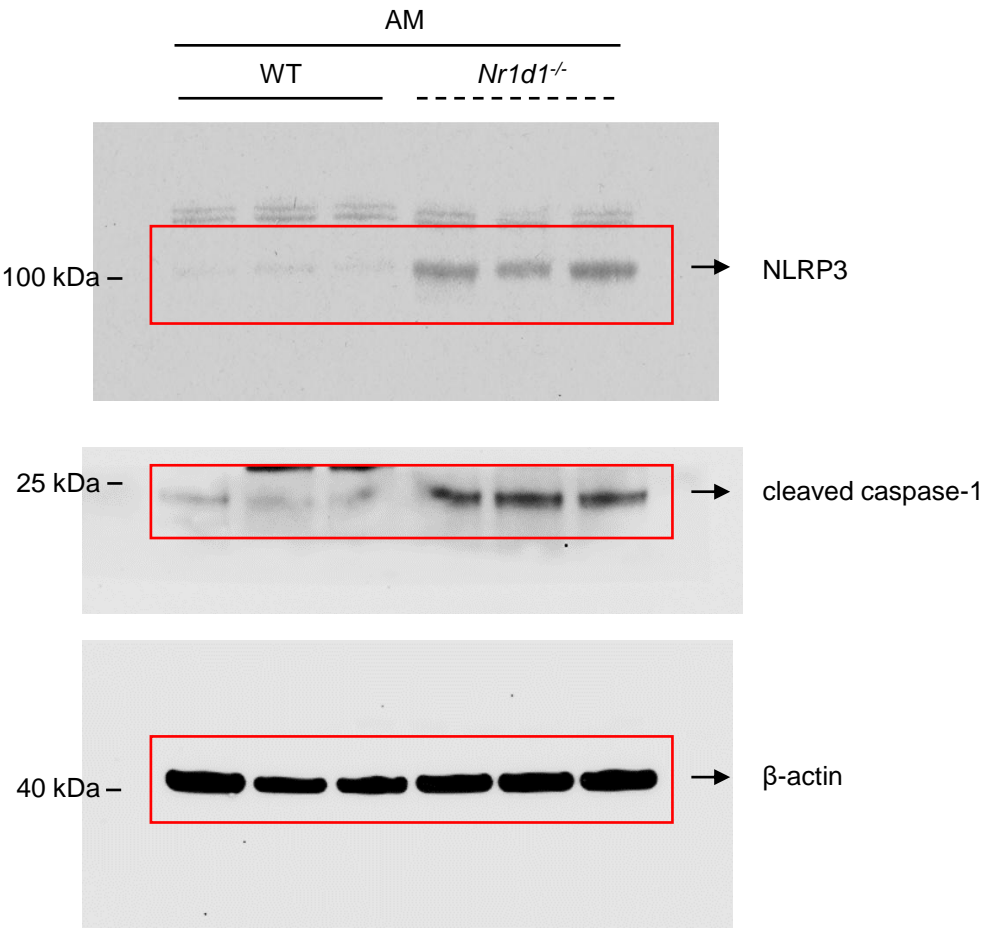

Fig. S6C

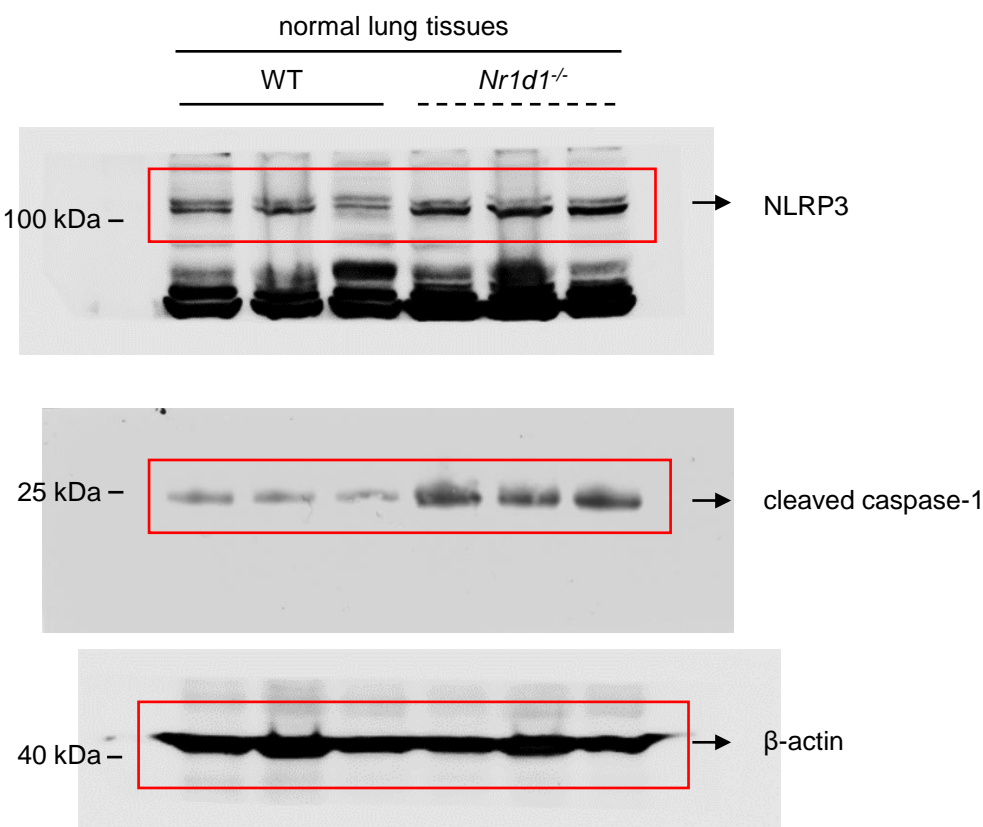

Supplement: Supplementary file 2 — Original western blot [file 41420_2023_1554_MOESM2_ESM.pdf]
